# Revealing Commercial Epoxy Resins’ Antimicrobial Activity: A Combined Chemical–Physical, Mechanical, and Biological Study

**DOI:** 10.3390/polym16182571

**Published:** 2024-09-11

**Authors:** Mario Rigo, Hamoun Khatami, Antonella Mansi, Anna Maria Marcelloni, Anna Rita Proietto, Alessandra Chiominto, Ilaria Amori, Annalisa Bargellini, Isabella Marchesi, Giuseppina Frezza, Francesco Lipani, Claudio Cermelli, Angelo Rossini, Marino Quaresimin, Michele Zappalorto, Alessandro Pontefisso, Matteo Pastrello, Daniele Rossetto, Michele Modesti, Paolo Sgarbossa, Roberta Bertani

**Affiliations:** 1 Department of Industrial Engineering, University of Padova, Via Marzolo 9, 35131 Padova, Italy; mario.rigo@unipd.it (M.R.); daniele.rossetto@unipd.it (D.R.); michele.modesti@unipd.it (M.M.); paolo.sgarbossa@unipd.it (P.S.); 2 Faculty of Engineering, Urmia University, 11km Sero Road, Urmia 5756151818, Iran; h.khatami@urmia.ac.ir; 3 Department of Occupational and Environmental Medicine, Epidemiology and Hygiene, Italian Workers’ Compensation Authority (INAIL), Via Fontana Candida 1, Monte Porzio Catone, 00078 Rome, Italy; a.marcelloni@inail.it (A.M.M.); a.proietto@inail.it (A.R.P.); a.chiominto@inail.it (A.C.); i.amori@inail.it (I.A.); 4 Department of Biomedical, Metabolic and Neural Sciences, Section of Public Health, University of Modena and Reggio Emilia, Via Campi 287, 41125 Modena, Italy; isabella.marchesi@unimore.it (I.M.); giuseppina.frezza@unimore.it (G.F.); 187569@studenti.unimore.it (F.L.); claudio.cermelli@unimore.it (C.C.); 5 Medical Services, Santa Lucia Foundation IRCCS, 00179 Rome, Italy; a.rossini@hsantalucia.it; 6 Department of Management and Engineering, University of Padova, Stradella S. Nicola 3, 36100 Vicenza, Italy; marino.quaresimin@unipd.it (M.Q.); michele.zappalorto@unipd.it (M.Z.); alessandro.pontefisso@unipd.it (A.P.); matteo.pastrello@phd.unipd.it (M.P.)

**Keywords:** commercial epoxy resins, mechanical properties, antibacterial activity, virucidal activity, surface properties characterization

## Abstract

In our continuing search for new polymer composites with antimicrobial activity, we observed that even unmodified epoxy resins exhibit significant activity. Considering their widespread use as starting materials for the realization of multifunctional nanocomposites with excellent chemical and mechanical properties, it was deemed relevant to uncover these unexpected properties that can lead to novel applications. In fact, in places where the contact with human activities makes working surfaces susceptible to microbial contamination, thus jeopardizing the sterility of the environment, their biological activity opens the way to their successful application in minimizing healthcare-associated infections. To this end, three commercial and widely used epoxy resins (DGEBA/Elan-TechW 152LR, **1**; EPIKOTE^TM^ Resin MGS^®^/EPIKURE^TM^ RIM H 235, **2** and MC152/EW101, **3**) have been investigated to determine their antibacterial and antiviral activity. After 24 h, according to ISO 22196:2011, resins **1** and **2** showed a high antibacterial efficacy (R value > 6.0 log reduction) against *Staphylococcus aureus* and *Escherichia coli*. Resin **2**, prepared according to the ratio epoxy/hardener indicated by the supplier (sample **2a**) and with 10% *w*/*w* hardener excess (sample **2b**), exhibited an intriguing virucidal activity against Herpes Simplex Virus type-1 and Human Coronavirus type V-OC43 as a surrogate of SARS-CoV-2.

## 1. Introduction

Healthcare-associated infections (HAIs) represent the most frequent and serious complication of healthcare, and they occur in different types of care settings, such as acute care hospitals, intensive care units, outpatient clinics, rehabilitation and long-term care facilities, outpatient dialysis facilities, etc. [1]. The European Centre for Disease Prevention and Control (ECDC) estimated that the total annual number of patients with at least one HAI in acute care hospitals in the European Union and European Economic Area countries was 4.3 million patients per year [2]. However, most HAIs are preventable and can be reduced by up to 70% through the application of effective Infection Prevention and Control (IPC) measures [3].

In the last decades, much scientific evidence has been collected regarding the role played by inanimate surfaces in the transmission of nosocomial pathogens, with particular attention to the objects more frequently touched by patients and healthcare personnel (High Touch Surfaces, HTSs) such as bedside tables, switches, push-buttons, computer keyboards, etc. [4]. International and national guidelines [5] recommended cleaning and disinfection by chemical germicides to reduce the level of microbiological contamination of environmental surfaces in hospital rooms. Unfortunately, several studies [6,7] have demonstrated that less than 50% of hospital room surfaces are adequately cleaned and disinfected by cleaning workers.

To prevent and control microbiological contamination on HTSs, some no-touch technologies (UV devices, hydrogen peroxide systems, cold plasma technology, etc.) have been developed over the years [8] as alternatives to conventional cleaning. More recently, self-disinfecting surfaces with incorporated antimicrobial substances have been proposed to support or replace either no-touch or conventional cleaning, and they represent an emerging research topic in continuous and rapid development. These innovative collective protective measures have several advantages over the other cleaning strategies because, following contact, they are able to kill most microorganisms, preventing their spread on other HTSs by healthcare personnel’s hands [9].

Since the COVID-19 pandemic, great efforts have been directed towards the design, creation, and characterization of self-disinfecting surfaces capable of killing/inhibiting the growth of microbes [10,11,12,13]. The limitation and control of the spread of microbial infections still represents an engaging scientific endeavor with a huge social impact. To this end, the incorporation of antimicrobial agents into polymers with intrinsic antimicrobial activity (chitosan, ε-Polylysine, Polymers Containing Quaternary Nitrogen Atoms or Aromatic or Heterocyclic Groups, etc.) has gained significant attention in recent years, driven by the increasing demand for materials with built-in protection against microbial colonization. Polymeric nanoparticles can kill microbes either through their contact-killing cationic surfaces (quaternary ammonium compounds, quaternary phosphoniums, or alkyl pyridiniums) or by releasing antimicrobial agents and antimicrobial peptides. The antibacterial activity of polycations depends on the ability of multiple charges to attach to and interact with the bacterial cell wall [14].

Epoxy resins have long been recognized as versatile materials with widespread applications in various industries, including aerospace, automotive, marine, and construction, due to their exceptional mechanical properties and ease of processing. Their unique chemical structure, characterized by epoxide groups, allows for cross-linking with a variety of curing agents, resulting in a three-dimensional network with remarkable strength, stiffness, and durability [14,15,16], together with good processability in injection and molding [17,18]. Owing to the tunable chemical properties of the epoxy resin networks, they are particularly suitable for preparing composites exhibiting improved characteristics, which can expand their use in new fields of application. Some recent intriguing examples are the introduction of multi-walled carbon nanotubes, graphene nanoplatelets, or graphene oxide in composites reinforced with carbon fibers to confer tunable mechanical and electrical properties [19,20]; the introduction of fluorine atoms to give superhydrophobic characteristics [21]; the dispersion of Ag-nanowires to improve electromagnetic interference for aerospace applications [22,23]; the compatibilization with layered silicates or POSS due to the polarity of the epoxy monomer to improve thermal and flame retardant properties [24,25]; or the incorporation of epoxy prepolymer and mercaptan in PMMA microcapsules to achieve self-healing epoxy composites [26]. The mechanical properties of epoxy resins, including tensile strength, modulus, fracture toughness, and fatigue resistance, are of paramount importance in determining the structural integrity and reliability of engineered components. Additionally, the interaction between mechanical loading and antimicrobial efficacy represents a novel aspect that warrants thorough investigation to ensure the compatibility and synergistic enhancement of these properties.

Composite epoxy resins with incorporated antimicrobial agents have been recently proposed as candidates in the quest for new materials with built-in protection against microbial colonization [27]. By integrating antimicrobial functionalities directly into epoxy matrices, researchers aim to develop novel composite materials capable of resisting microbial adhesion, proliferation, and biofilm formation, thus extending their service life and enhancing their suitability for diverse applications. One of the most important applications of epoxy resins is in endodontics, for which suitable mechanical and chemical compatibility must be accompanied by antimicrobial efficacy with the elimination of microorganisms and prevention of re-contamination, together with their ability to improve tissue regeneration [28].

Within our ongoing research on new polymer-based composites suitably modified to exhibit antimicrobial activity, particularly based on epoxy resins [27], we found that even unmodified commercial resins show significant antimicrobial activity. This prompted us to study this aspect in more depth, selecting three commercial epoxy resins (DGEBA/Elan-TeckW 152LR; EPIKOTE^TM^ Resin MGS^®^/EPIKURE^TM^ RIM H 235 and MC152/W101) among those most widely used. After a thorough characterization in terms of chemical composition and physical and mechanical properties, we undertook a comprehensive investigation of their antimicrobial in vitro activity. The latter addressed both their antibacterial and antiviral in vitro action in view of their possible use in the creation of HTSs to be placed in healthcare settings, where they are highly susceptible to microbial contamination. Their use, either neat or as nanocomposites, may lead to the prevention or reduction of healthcare-associated infections, which usually result in undesired medical complications.

## 2. Materials and Methods

### 2.1. Materials

**Figure 1:**
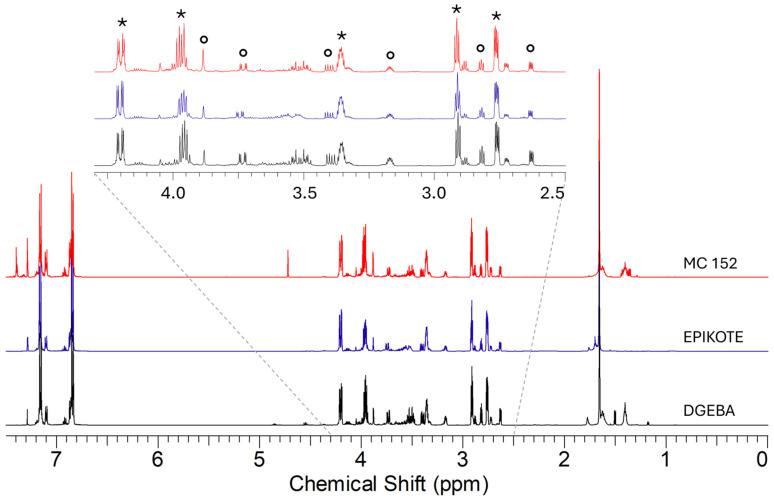
^1^H NMR spectra in CDCl_3_ of the epoxy precursors. The signals are highlighted by different symbols: * bis-phenol-A epichlorohydrin; ∘ bis-phenol-F epichlorohydrin.

**Figure 2:**
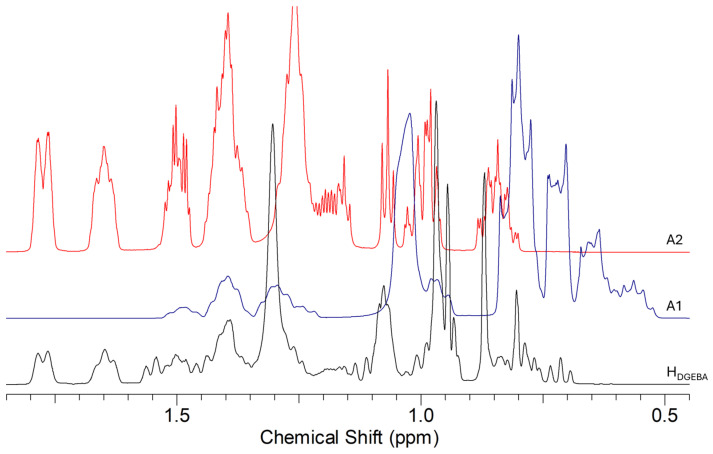
^1^H NMR spectra in CDCl_3_ in the range 0.5–2.0 ppm of the Elan-TechW 152LR (H_DGEBA_) hardener and the amines: A1 = 3-aminomethyl-3,5,5-trimethylcyclohexylamine; A2 = 4,4′-methylenebis-cyclohexylamine.

Three commercial epoxy resins were prepared starting from the following polymer precursors and the corresponding hardeners: (i) Diglycidyl ether of bisphenol A epoxide (DGEBA, Elan-Tech EC157) and a mixture of cycloaliphatic amines (Elan-TechW 152LR), both supplied by Elantas (Elantas Europe s.r.l., Collecchio, Parma, Italy), (ii) EPIKOTE^TM^ Resin MGS^®^ and EPIKURE^TM^ RIM H 235, both supplied by Hexion (Hexion, Columbus, OH, USA) and (iii) MC152 and W101, both supplied by Elantas. The epoxy resins were prepared according to the resin/curing agent ratios indicated by the suppliers and in the presence of about 10% *w*/*w* excess of curing agent for (i) and (ii). (Table 1) ^1^H NMR and FTIR analyses showed that DGEBA, EPIKOTE^TM^, and MC152 contained mixtures of bis-phenol A and bis-phenol F epichlorohydrin as epoxy precursors, as shown in Figure 1 and Figure S1, while the curing agents were mixtures of cyclohexylamines such as 3-aminomethyl-3,5,5-trimethylcyclohexylamine and 4,4′-methylenebis(cyclohexylamine) (Figure 2 and Figure S2). As for (iii), according to MSDS, the epoxy precursors were bisphenol F-epichlorohydrin, bisphenol A-epichlorohydrin, 2,2-bis-[4-(2,3-epoxypropoxy)phenyl]-propane, and 1,6-bis(2,3-epoxypropoxy)hexane, while the curing agent contained 3-aminomethyl-3,5,5-trimethylcyclohexylamine. In the epoxy precursor of (iii), there was also a high content (about 60% *w*/*w*) of inorganic fillers (TiO_2_, SiO_2_, CaCO_3_, MgCO_3,_ and Al_2_O_3_; Figure S4), as proven by ESEM/EDX determinations.

### 2.2. Preparation of the Epoxy Resins

The reactions were performed in a flask by the addition of the curing agent to the epoxide component, according to the ratios indicated in Table 1. The mixtures were mechanically stirred at room temperature for about 15 min. A final degassing step was necessary prior to casting the resin in the silicon molds, where resins were cured for 48 h at room temperature; then, the samples were demolded before post-curing at 70 °C for 8 h for **1** and **2**. For sample **3a**, more elaborate preparation was necessary to minimize the entrapment of air in the blend due to the high viscosity of the epoxy (about 8 Pas). In detail, the epoxide component was pre-heated at 50 °C while being degassed at about −1 atm for 3 h and 30 min. Then, the curing agent was added, and the mixture was mechanically stirred under vacuum for 5 min. After casting into silicone molds, the nanocomposite was cured for 48 h at room temperature and then at 60 °C for 10 h. Through DSC determinations, the epoxy resins were shown to be completely cured. The **1b** and **2b** samples were prepared to evaluate the influence of the hardener excess on the antimicrobial activity of the materials.

### 2.3. Characterization Techniques

Nuclear Magnetic Resonance. The starting materials (the epoxy precursors DGEBA Elan Tech EC157, EPIKOTE^TM^ Resin MGS^®^, and MC152; the hardeners Elan-TechW 152LR, EPIKURE^TM^ RIM H 235, and W101, together with 3-aminomethyl-3,5,5-trimethylcyclohexylamine and 4,4′-methylenebis-cyclohexylamine) were characterized by multinuclear NMR experiments using a 600 MHz Bruker Avance spectrometer (Bruker Italia s.r.l., Milano, Italy) equipped with a Prodigy cryoprobe; ^1^H and ^13^C assignments of each product were determined by 1D conventional and 2D COSY, HSQC, and HMBC experiments.

Fourier Transform Infrared Spectroscopy. ATR-FTIR spectra were collected with the Spectrum 100 Perkin Elmer instrument (Perkin Elmer Italia s.p.a., Milano, Italy) at 20 °C, 35–45% RH (32 reflections, diamond/ZnSe crystal). The samples of 2 mm thickness were prepared according to the method outlined in Section 2.2.

The point of zero charge (PZC) was determined using the Anton Paar SurPASS 3 (Anton Paar Italia s.r.l., Rivoli, Torino, Italy) through the analysis of the zeta potential at the solid/liquid interface at variable pH (from 5 to 10 every 0.5 pH unit).

Environmental Scanning Electron Microscopy. The microanalyses and morphologies of the samples were studied by E.S.E.M. (Quanta 200 FEI-XRF embedded, FEI Italia s.r.l., Milano, Italy). The samples were observed directly on the surface, and cross-sections were obtained by brittle fracture at the temperature of liquid nitrogen. Images and data are reported in Figure S4 and Table S1.

Density measurements were carried out by hydrostatic weighing with an electronic SD-200L densimeter (Oakland Instrument Corporation, Shakopee, MN, USA).

Thermogravimetric and DSC Analyses. TGA was performed under N_2_ atmosphere from room temperature on a TA Instrument SDT Q600 thermogravimetric analyzer (TA Instruments, Sesto San Giovanni, Milano, Italy), with a heating rate of 10 °C/min from 25 °C to 600 °C (or 800 °C for **3**). DSC determinations were carried out with a differential scanning calorimeter (TA Instruments DSC Q200, New Castle, DE, USA). Samples (5–10 mg) were cyclically heated and cooled between −50 °C (or 0 °C or 20 °C) and 250 °C under a nitrogen atmosphere using 10 °C/min as heating and cooling rates; the first heating cycle was used to remove the effect of the prior thermal history of the resin samples. The glass transition temperature (T_g_) was determined as the temperature corresponding to the midpoint of the step in the third cycle.

DMA tests were carried out with a Q800 (TA instruments, New Castle, DE, USA) analyzer. The samples (10 mm × 80 mm × 3 mm) were tested with a single cantilever clamp with a fixed strain of 20 μm applied at 1 Hz of frequency over the temperature range between 35 and 120 °C at a fixed heating rate of 3 °C/min. The glass transition temperature (T_g_) was identified by the peak in the loss modulus curve.

Mechanical Properties. Tensile tests on dog-bone (DB) specimens were carried out taking advantage of an electrodynamic axial testing machine, StepLab EA05 (StepLab Resana, Treviso, Italy), equipped with a 10 kN-class 1 AEP transducer type TC-4 using a crosshead speed equal to 1 mm/min and with an Epsilon 3542-025m-100-st axial extensometer (StepLab Resana, Treviso, Italy). The specimen geometry was chosen according to ISO 527-2 [30]. Five specimens were tested for each sample to obtain statistically representative results. In all the considered cases, failure took place in the gauge length of the specimens. Fracture tests were carried out following the ASTM D5045-99 guidelines on compact tension (CT) specimens using the same equipment as tensile tests [31]. The specimens were pre-cracked by manually tapping a fresh razor blade with a hammer, and only those specimens exhibiting straight cracks of length in the range 10–30 mm were retained. Five specimens for each sample were then tested with a crosshead speed equal to 10 mm/min.

Antibacterial Activity. The antibacterial activity of the epoxy resins was evaluated following ISO 22196:2011 [32]. A Gram-negative (*Escherichia coli*, ATCC^®^ 8739) and a Gram-positive bacterium (*Staphylococcus aureus*, ATCC^®^ 6538P) were used for in vitro testing. The strains from the frozen stock cultures were transferred to screw-capped tubes containing Nutrient Agar medium and incubated at 35 ± 1 °C for 18–20 h. Subsequently, one loop of each bacterial strain was transferred into tubes containing Nutrient Broth (NB) diluted 1:500 in deionized water and incubated overnight. Prior to inoculation, the bacterial suspensions were placed into fresh NB diluted 1:500 and incubated in an orbital shaker at 35 ± 1 °C until reaching exponential-phase growth. Lastly, bacteria were centrifuged (2500 rpm, 10 min) and then resuspended in phosphate-buffered saline (PBS). The number of bacteria was spectrophotometrically adjusted to reach the target bacterial concentration of about 6 × 10^5^ Colony-Forming Units (CFUs)/mL for use as test inoculum. Epoxy resin test specimens (standard size 50 mm × 50 mm) were placed on sterile Petri dishes and then sterilized using UV Germicidal Lamps (254 nm) for 30 min on each side. The bacterial inoculum (400 µL) was deposited on the epoxy resin specimens and on polypropylene (PP) specimens used as control. All inoculated samples were covered with sterile polyethylene film (40 mm × 40 mm) and incubated at 35 ± 1 °C for 24 h in a plastic box containing paper saturated with water to maintain high relative humidity levels not less than 90%. The antibacterial activity of the epoxy resins and PP specimens was evaluated at time zero (T0) immediately after the deposition of bacterial inoculum and at 24 h (T24) of incubation. After, the specimens were transferred into conical tubes with 10 mL of neutralizing solution (D/E neutralizing broth, BD Difco^TM^, Franklin Lakes, NJ, USA) and were vortexed for 60 s to detach bacteria. Serial dilutions of this solution were inoculated for inclusion on Plate Count Agar (PCA, Oxoid Ltd., Basingstoke, Hampshire, UK) and then incubated at 35 ± 1 °C for 24–48 h. Finally, the bacterial colonies on each plate were enumerated, the number of CFUs/cm^2^ was calculated, and the bacterial counts were converted into decimal logarithms.

Antiviral Activity. The virucidal activity was tested against three pathogenic human viruses: Herpes Simplex Virus type-1 (HSV-1) and AdenoVirus type-5 (ADV-5), which were obtained from clinical isolates, identified with monoclonal antibodies, and adapted for in vitro growth by serial passages on VERO cell cultures; and Human CoronaVirus OC43 (HCoV-OC43), purchased from the American Type Culture Collection (ATCC VRI-558). Batches of each virus were prepared, titrated on the suitable cell line, and kept frozen at –80 °C until they were used for the experiments. HSV-1 and AdV-5 were cultivated in the VERO cell line. The cells were grown in RPMI 1640 medium with added ciprofloxacin (20 mg/mL), penicillin (100 U/mL), streptomycin (100 μg/mL), L-glutamine (2 mM), and fetal bovine serum (FBS) at 5% or 10%, depending on whether maintenance or growth medium was required, respectively. Bi-weekly passages in fresh medium maintained the cell lines. D54-MG cell line, a transformed human astrocyte cell line from an astroglioma, was used to cultivate HCoV-OC43 in Dulbecco’s Modified Eagle Medium (DMEM) supplemented with ciprofloxacin, L-glutamine, penicillin, streptomycin, and FBS as previously described for RPMI medium. Two different protocols were used to test the virucidal activity: protocol 1 had the aim of verifying the virucidal activity of resins by contact, while protocol 2 was set to assay whether the resins release virucidal molecule(s) in the medium. In both cases, prior to use in each experiment, resin coupons (2 × 1 cm) were exposed to UV light (15 min/side) to sterilize them. For Protocol 1 (contact test), the resin coupons were soaked in 2 mL of a viral suspension of known concentration, vortexed for 30 s, and then incubated for 1 h and 24 h at room temperature. For Protocol 2 (release test), the coupons were soaked in 1 mL of maintenance medium for 24 h with continuous shaking. After 24 h, the coupon was discarded, and the supernatant was incubated with the viral suspensions for 1 h and 24 h. In both cases, at the end of the incubation time, the residual viral load was quantified by end-point titration. Each sample was serially diluted in culture medium, and each 10-fold dilution was seeded on the suitable cell cultures (VERO cells for AdV-5; HSV-1 and D54MG for HCoV-OC43) in a 96-well tissue culture plate 24 h after cell preparation. Plates were incubated for 3 days at 37 °C in a CO_2_ incubator, and then the cell cultures were observed under an inverted light microscope to assess the cytopathic effect (CPE) typical of the different viruses. The highest dilution showing CPE was used to determine the viral titer, expressed as the dose infecting 50% of the cell cultures (TCID50), using Reed and Meunch’s formula [33]. In order to determine whether the resins release any toxicity on the cell cultures used for virus growth, the cytotoxicity test LDH assay was used (Roche Diagnostics, S.p.A., Monza, Italy). For this purpose, 100 µL of each sample from the release experiments was processed according to the manufacturer’s instructions.

Statistical analysis. All experiments were performed in at least three independent replicates. Mean ± Standard Deviation (SD) was calculated for each experiment, and the data were subjected to Student’s *t*-test, one-way analysis of variance (ANOVA), and the Bonferroni post hoc test or Mann–Whitney test when appropriate. The statistical significance of the experimental results was calculated by GraphPad Prism software (version 10, GraphPad Software, San Diego, CA, USA).

## 3. Results

### 3.1. Characterization of the Epoxy Resins and Precursors

Even though they are very complex due to the presence of many undisclosed additives, we considered it fundamental to start by understanding the chemical nature of the industrial formulations used in the preparation of the three commercial resins. In doing so, we necessarily focused on the most abundant components of both the epoxy precursors and hardeners.

In Figure 1, the ^1^H NMR spectra of the epoxy precursors are reported: it could be observed that they contain bis-phenol-A and bis-phenol-F-epichlorohydrin in different ratios (about 1:0.25 for DGEBA, 1:0.20 for EPIKOTE, and 1:0.33 for MC152, respectively), together with the characteristic components of the formulation.

As for the hardeners, in Figure 2, the ^1^H NMR spectrum of Elan-TechW 152LR is reported, together with those of 3-aminomethyl-3,5,5-trimethylcyclohexylamine and 4,4′-methylenebis-cyclohexylamine: both amines are present in the mixture (about in the ratio 1:1.25, respectively), together with other additives characteristic of the industrial product, whose presence justifies the small differences in chemical shifts due to pH modification.

The presence of both amines in the hardeners has also been confirmed in the FTIR spectra (signals at 1475 and 1376 cm^−1^ and shoulders; Figure S2) and by HPLC-MS determinations, where the signals at *m*/*z* 171 corresponding to the molecular ion of protonated 3-aminomethyl-3,5,5-trimethylcyclohexylamine and at *m*/*z* 249 of the alkylated derivative NH_2_-Cy-CH_2_-Cy-CH-(CH_2_)_3_H^+^ of 4,4′-methylenebis-cyclohexylamine were present.

**Figure 3:**
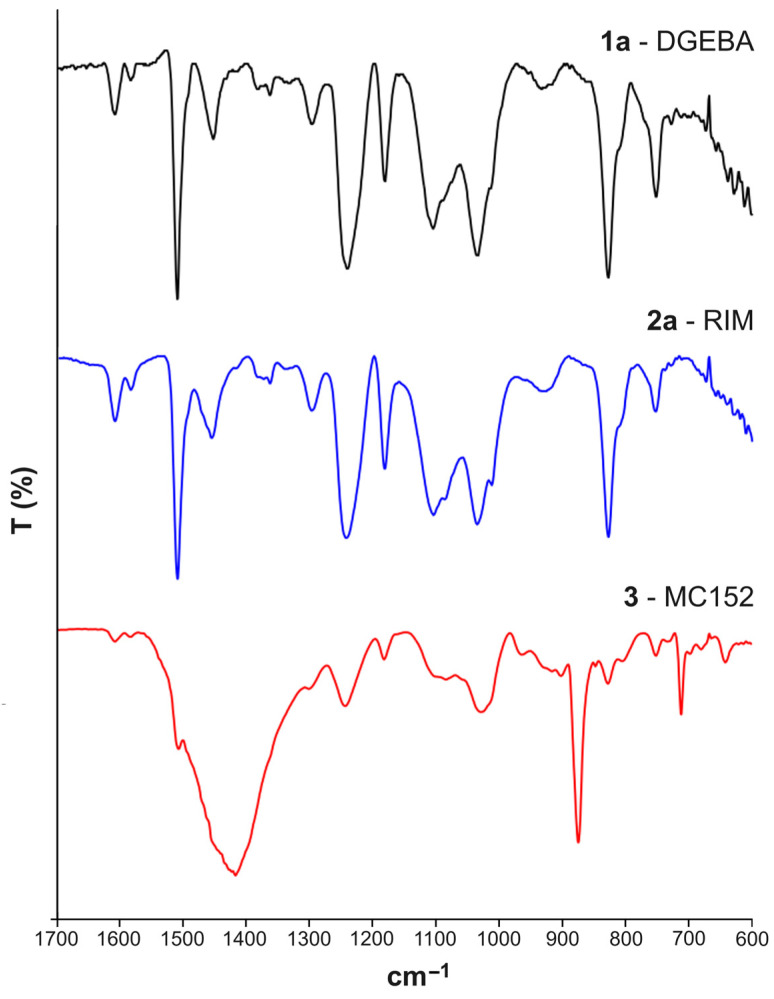
FTIR spectra of the samples **1a**, **2a**, and **3** in the range 1700–600 cm^−1^.

As for the resins, both the FTIR-ATR of samples 1 and 2 in the mid-IR (mIR) region showed the following signals: at 3500 cm^−1^ (OH stretching); in the range 2960–2868 cm^−1^ (symmetric and asymmetric stretching C-H of CH_2_); at 1607 cm^−1^ (stretching C=C of aromatic rings); at 1509 cm^−1^ (stretching C-C of aromatics); at 1229 and 1035 cm^−1^ (stretching C-O-C of ethers); at 827 cm^−1^ (stretching C-O-C of the oxirane group); and at 753 cm^−1^ (rocking CH_2_), according to the literature (Figure 3) [34]. It is of note that the signals at 831 and 829 cm^−1^ of the oxirane group were significantly more intense in the epoxy prepolymers DGEBA, Elan Tech EC157, and EPIKOTE^TM^ Resin MGS^®^ (Figure S1) compared with the corresponding **1** and **2** polymers. It is also of note that no difference can be observed in the mIR spectra for the samples prepared in the presence of an excess of hardener. As for the FTIR-ATR spectrum of sample **3**, the strong signals at 1417, 875, and 715 cm^−1^ typical of calcite were observed in addition to the absorptions at 1243, 1028, 829, and 752 cm^−1^.

The thermogravimetric analyses showed the T_onset_ at about 330 °C for compounds **1** and **2**, while for **3**, the thermal degradation processes started at 319 °C, followed by a second weight loss at 706 °C due to the decarboxylation of carbonates. Thermogravimetric determinations (Figure S3) indicated that T_onset_ for **1a** and **2a** resulted in similar values observed for the corresponding **1b** and **2b**. The pyrolysis processes involved one stage with a maximum degradation rate at about 368 °C for **1** and **2** samples and at 349 °C for **3** [35]. The thermogravimetric analysis of **3** showed a first weight loss of 4% at 90 °C, a second process at 319 °C with a weight loss of 24%, and a final process at 709 °C with a weight loss of 26%, reasonably due to decomposition of the inorganic fillers.

DMA measurements (Figure S3) allowed the detection of the glass transition temperature T_g_ from the peak of the loss modulus and tanδ curves [36], achieving quite similar results for **1a** and **2a** but lower values in the case of **3**, for which the higher value of storage modulus, which strongly depends on the specimen dimensions (about 7000 MPa compared with about 2000 MPa for **1** and **2**) was due to the high filler content and not to the improved reticulation of the polymeric matrix.

### 3.2. Mechanical Properties

**Figure 4:**
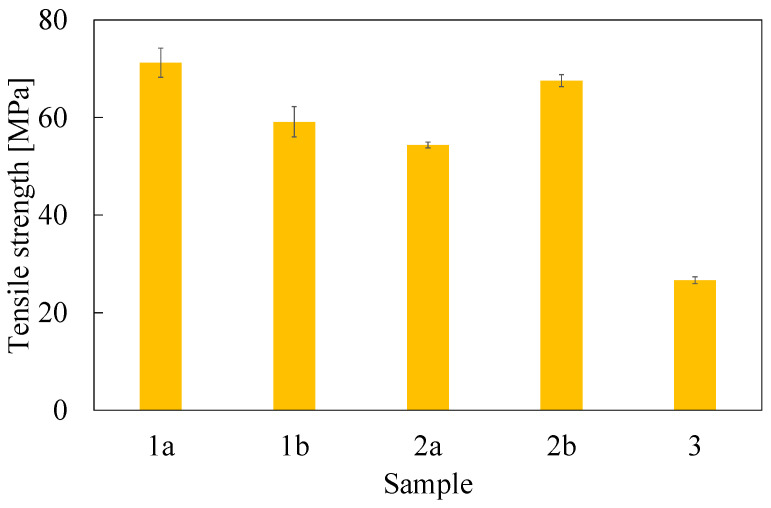
Ultimate tensile strength evaluated from dog-bone specimens. Error bar is determined as standard deviation.

**Figure 5:**
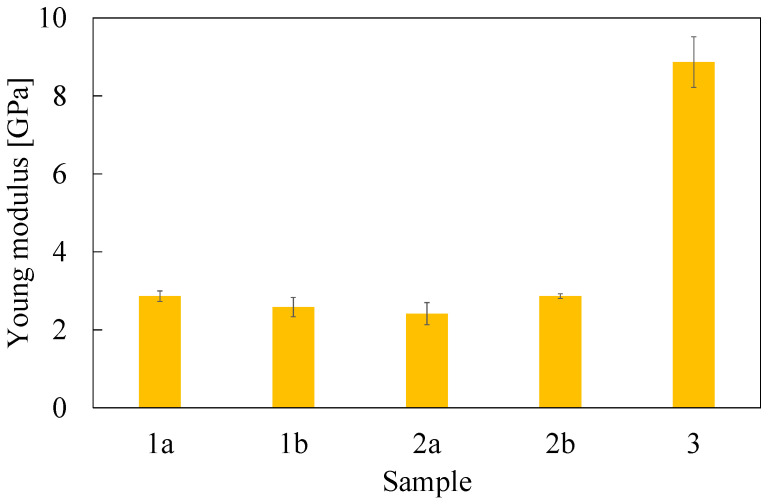
Young modulus evaluated from dog-bone specimens. Error bar is determined as standard deviation.

**Figure 6:**
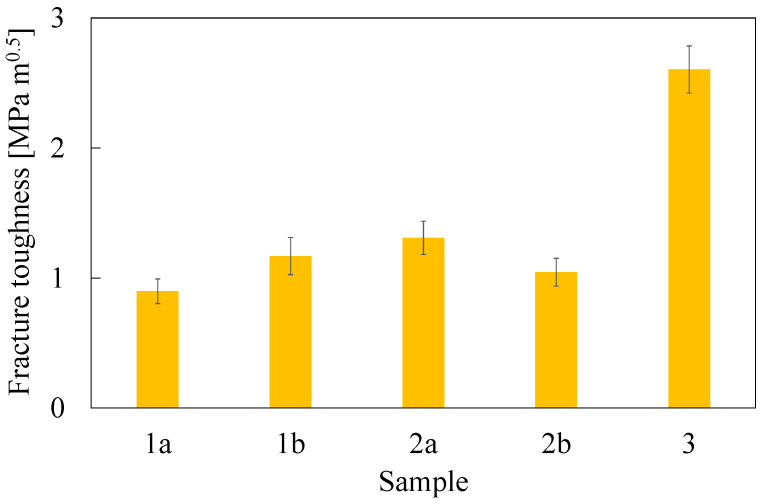
Fracture toughness evaluated as K_IC_ from compact-tension specimens. Error bar is determined as standard deviation.

**Figure 7:**
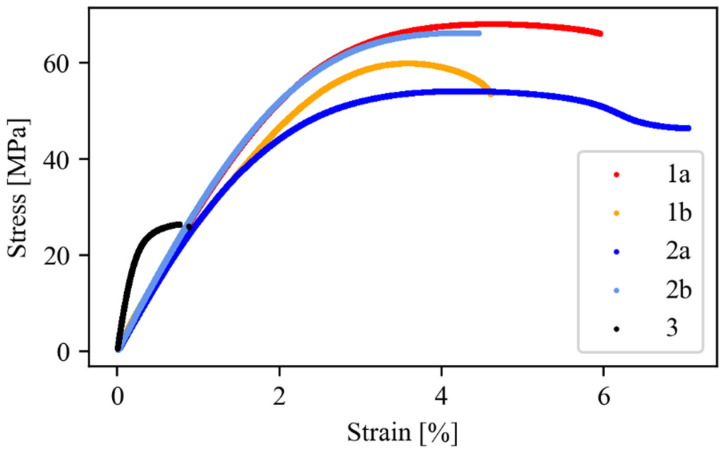
Stress–strain curves obtained from dog-bone specimen testing. For each material, a representative curve is plotted.

**Figure 8:**
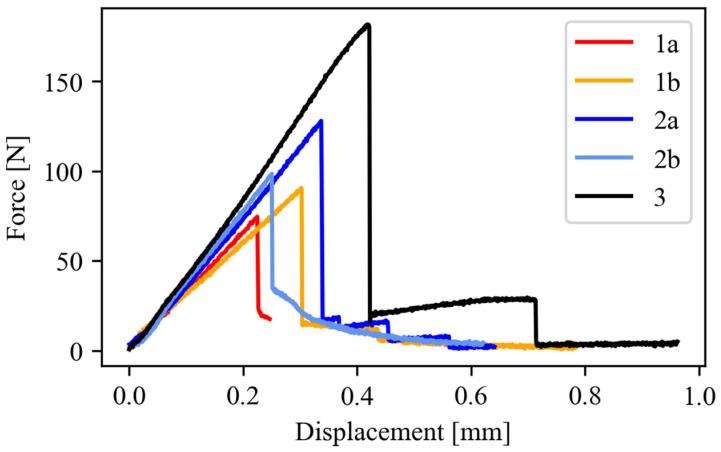
Force–displacement curves obtained from CT specimen testing. For each material, a representative curve is plotted.

Tensile and fracture toughness properties are reported in Table 2 and Figure 4, Figure 5 and Figure 6. Tensile properties for samples **1a**, **2a**, and **3** were consistent with those reported in the manufacturers’ datasheets. Concerning tensile strength, samples **1a** and **2a** exhibited remarkably high performance in comparison with sample **3**. Differently, in terms of Young modulus and fracture toughness, sample **3** significantly outperformed the former. The fact that sample **3** differed significantly from the others is an expected outcome of nanomodification, which raises the Young modulus and the fracture toughness at the cost of reduced tensile strength [27]. Another side effect was the high viscosity of the epoxide component used for sample **3**, which was about one order of magnitude higher than the others. This remarkable difference limited the applicability of sample **3** to components manufactured by casting, while **1a** and **2a** were suitable for vacuum-assisted resin transfer molding, as well. Regarding the effect of the curing agent excess, appreciable differences were detected both in samples **1** and **2**, even though the effect was not consistent across the two materials. In the case of sample **1b**, the curing agent excess led to a decrease in the average tensile strength and Young modulus of 17% and 10%, respectively, while the average fracture toughness increased by 30%. In the case of material **2**, the average tensile strength and the Young modulus both increased by 24% and 19%, respectively, while the average fracture toughness decreased by 20%. It is worth pointing out that the standard deviation reported in Table 2 supports the statistical soundness of the abovementioned considerations, marking the performance of the materials obtained by hardener excess as statistically different from that obtained by using a correct stoichiometric ratio. On average, it seems that the hardener excess reduced the strain at which the maximum tensile strain is detected (Figure 7). However, the overall shape of the curves is preserved, suggesting that the hardener excess may not affect the structural configuration of the material. Considering fracture tests (Figure 8), all materials exhibited linear-elastic behavior up to fracture, with some stick-slip features. Such behavior is common in epoxy resins and nanomodified epoxies, given their glassy nature. Out of these results, three considerations can be drawn: (i) an excess of curing agent affected more than proportionally the investigated mechanical properties; (ii) the fracture toughness trend was opposite to those of tensile properties; (iii) different epoxy systems were affected in different ways by an excess of curing agent, with the hardener in excess behaving as a plasticizer in the case of **1**, thus reducing tensile strength and Young modulus but increasing fracture toughness, while in the case of **2**, the excess of curing agent increased the Young modulus and decreased fracture toughness.

### 3.3. Antibacterial Activity

**Figure 9:**
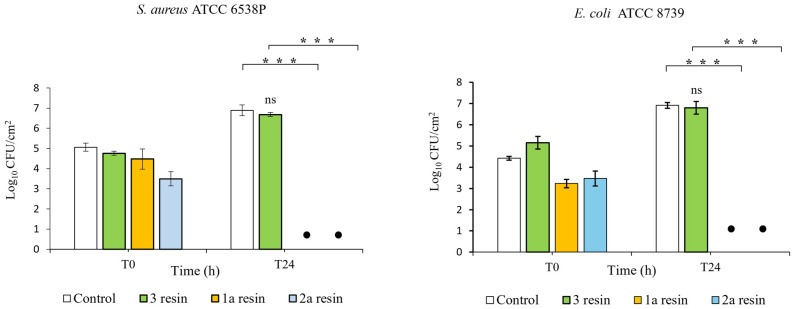
Bactericidal activity of three epoxy resins towards *S. aureus* and *E. coli*: MC152 = **3**; DGEBA = **1a**; EPIKOTE = **2a**. The number of bacteria was quantified immediately after inoculation (T0) and after 24 h of incubation (T24) compared to control (PP). The mean value of at least 3 experiments ± SD is presented. Differences with respect to controls were marked with asterisk (*) and defined as statistically significant at *** *p* < 0.001; not significant (ns). • No bacteria colonies detected.

**Figure 10:**
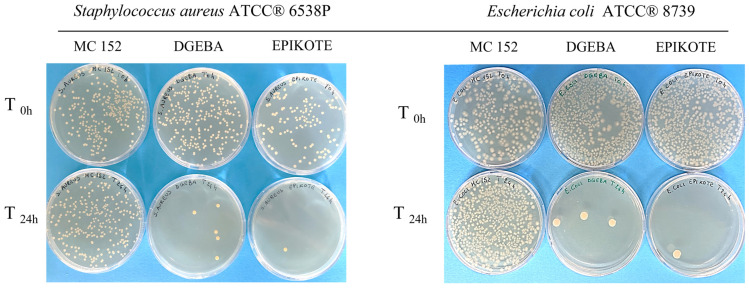
*Staphylococcus aureus* and *Escherichia coli* colonies at T0 and after 24 h contact with the three resins: **1a** (DGEBA), **2a** (EPIKOTE), and **3** (MC152).

In order to assess the antibacterial activity of three epoxy resins, we performed in vitro tests according to ISO 22196:2011. It specifies a method of evaluating the antibacterial activity of plastics and other non-porous surfaces. In this study, the results were obtained by considering the calculation formula established by the standard. The R value was determined by calculating the average reduction value of the number of bacterial cells on the epoxy resin specimen compared to the control (PP) according to the R = (U_t_ − U_0_) − (A_t_ − U_0_) = U_t_ − A_t_ relationship, where U_0_ is the average of the common logarithm of the number of viable bacteria in CFUs/cm^2^ recovered from the PP sample immediately after inoculation (T_0_); U_t_ is the average of the common logarithm of the number of viable bacteria in CFUs/cm^2^ recovered from the PP specimens after 24 h; and A_t_ is the average of the common logarithm of the number of viable bacteria in CFUs/cm^2^ recovered from epoxy resins specimens after 24 h. Log-transformed data were used for the analysis of bacterial counts; the antibacterial activity was attributed to those samples able to determine a reduction value (R) in the number of bacterial colonies ≥ 2 logarithmic units per cm^2^ of surface (Log 10 CFUs/cm^2^) within a contact time of 24 h. The tests were carried out on three commercial epoxy resins as yet unexplored in terms of antimicrobial properties, showing that **1** and **2** epoxy resins were able to reduce the number of bacterial cells after a 24 h incubation period (Figure 9 and Figure 10).

**1a** and **2a** epoxy resins showed a high antibacterial efficacy (R value > 6.0 log reduction) with very similar mean reduction values against *E. coli* (R = 6.92 and R = 6.89, respectively) and *S. aureus* (R = 6.89 and R = 6.68, respectively). These results were statistically significant (*p* < 0.001) when compared to those of control samples (PP).

As for epoxy resin **3** samples, the results show that this resin has no bactericidal effect against the reference bacterial strains. In fact, when this resin was tested in vitro, the average reduction values for *S. aureus* ATCC 6538P and for *E. coli* ATCC 8739 were very low: R = 0.21 and R = 0.13, respectively.

The **1b** resin samples prepared with an increased amount of hardener were then tested in vitro (ISO 22196:2011) to assess whether the increased amounts of amines could enhance antibacterial efficacy against the reference bacterial strains. Finally, the results obtained show that mean values of microbial load reduction on the **1a** and **1b** resin samples were very similar and that the difference between these values was not statistically significant.

### 3.4. Antiviral Activity

**Figure 11:**
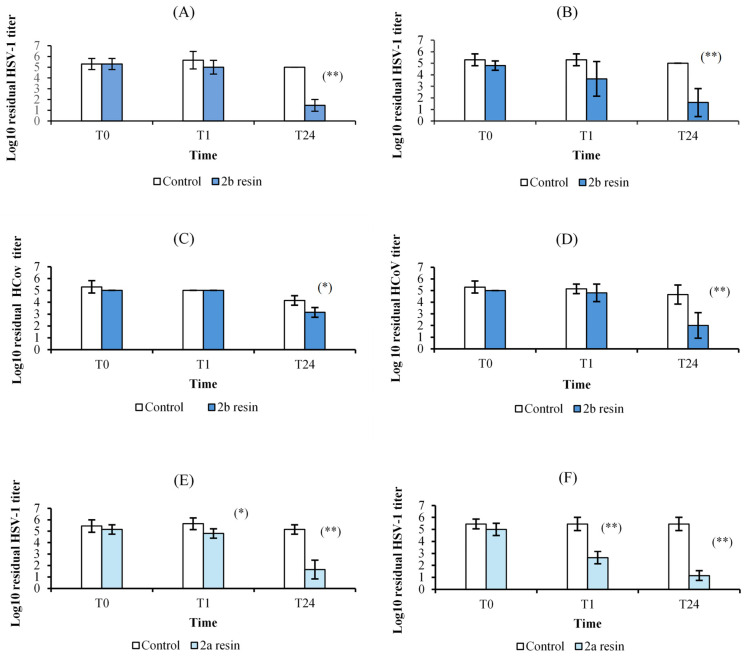
Virucidal activity of the **2a** (EPIKOTE) and **2b** (EPIKOTE with an excess of curing agent) resins against HSV-1 and HCoV-OC43 immediately after inoculation (T0) and after 1 h (T1) and 24 h (T24) of incubation compared to controls, according to the two different experimental protocols. (**A**) **2b** resin against HSV-1, contact test; (**B**) **2b** resin against HSV-1, release test; (**C**) **2b** resin against HCoV-OC43, contact test; (**D**) **2b** resin against HCoV-OC43, release test; (**E**) **2a** resin against HSV-1, contact test; and (**F**) **2a** resin against HSV-1, release test. The mean value of at least three experiments ± SD is presented. Differences with respect to controls were marked with an asterisk and defined as statistically significant at * *p* < 0.05 and ** *p* < 0.01.

**Figure 12:**
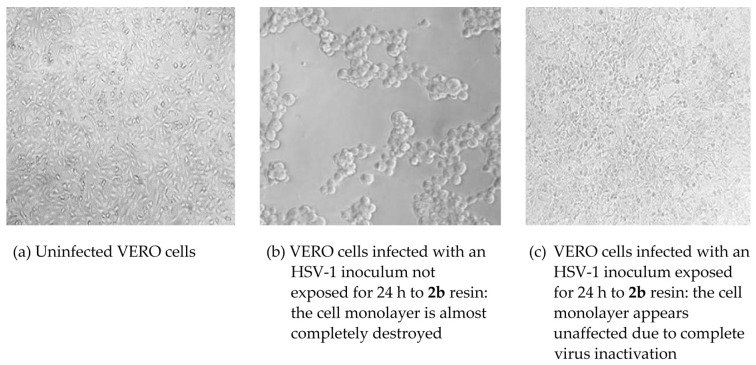
Virucidal activity of resin **2b** against HSV-1. The evaluations were carried out according to the procedure described in Section 2.

The **2a** and **2b** resins gave the most promising results in terms of virucidal activity (Figure 11A–F and Figure 12). Sample **2b** showed a remarkable inactivating activity against HSV-1 and, to a lesser extent, against HCoV-OC43, by both contact and release protocols, although the inhibition is greater by release. In addition, this activity is time-related. The viral titer reduction against HSV-1 was 0.7 Log after 1 h and 3.5 Log after 24 h following the contact protocol (Figure 11A) and 1.7 Log and 3.4 Log in the release test (Figure 11B); for HCoV-OC43, a significant reduction was obtained only after 24 h incubation, both by contact (1 Log reduction, Figure 11C) and release (2.7 Log, Figure 11D). Sample **2a** showed higher anti-HSV-1 activity than **2b** resin but no activity against HCoV-OC43. The experiments by contact (Figure 11E) showed a 0.8 Log and 3.5 Log reduction of HSV-1 titer after 1 h and 24 h, respectively, while the reductions were 2.8 Log and 4.3 Log in the release studies (Figure 11F). Both **2a** and **2b** samples had no activity against AdV-5. The other tested resins (**1a**, **1b**, and **3**) did not display any significant antiviral activity. The LDH studies carried out on the samples from the release experiments showed that no resin had cytotoxic activity.

## 4. Discussion

While the chemical and mechanical properties of the epoxy resins **1**–**3** agreed with the technical data sheets, and more in general with the literature, which justifies their widespread applications [37], the possible antimicrobial activity of their surface, to our knowledge, has never been investigated before. The latter is of particular interest nowadays since these materials could represent an easily available and inexpensive resource in the prevention of the spread of infections, reducing or avoiding the use of chemicals for sanitization purposes (e.g., chlorine, hydrogen peroxide, sodium hypochlorite, or ethanol). Moreover, the information obtained can help us understand the antimicrobial activity of composite systems obtained by the modification of resins **1** and **2**.

The antimicrobial activity of surfaces could be, in principle, based on two different mechanisms involving the release of antimicrobials from surfaces or the ability of surfaces to kill adhering bacteria directly upon adhesion without the release of antimicrobials, even if the exact antibacterial activity mechanism of a definite agent against specific microorganisms has not been completely elucidated [27,37,38].

In this study, we followed ISO 22196:2011, which is the most widely used method in the industry for evaluating the antibacterial activity of treated plastics and other non-porous surfaces. It is considered the gold standard, being capable of evaluating the potential biocidal efficacy of new materials after direct contact between the surface and a bacterial suspension at a known concentration within 24 h [39]. The above data showed that the **1** and **2** resins exhibited a relevant antibacterial in vitro activity either against *E. coli* or *S. aureus*. In addition, resin **2** showed a promising virucidal activity on Herpes Simples Virus type 1 and, limited to the formulation with an excess of hardener, HCoV-OC43 as a surrogate of SARS-CoV-2. In order to evaluate the virucidal activity of epoxy resins, we chose to perform a biological test on cell cultures because, although it is less sensitive than the detection and quantification of nucleic acid, it is the only test that provides results on the infectivity of residual virus [40]. In addition, all antiviral assays were carried out on complete cell culture media with fetal bovine serum and other organic material, thus simulating the soiled condition that we can find on real environmental surfaces. Finally, it is not surprising that the best results were obtained against HSV-1 and HCoV-OC43, which are enveloped viruses with limited resistance outside the host, while Adv-5 may persist as infectious on environmental surfaces for up to several months and shows high resistance to disinfection [41]. We are currently investigating the mechanism(s) of this antiviral activity. The finding of a marked virucidal effect not only by contact with the resins but also by release suggested that we should search for the presence of antiviral molecule(s) in the medium in which the resin blocks were left to incubate for 24 h. Thus, we carried out releasing experiments with **1a**, **2a**, and **2b** by putting two samples with 1 cm × 2 cm dimensions in PBS buffer (4 mL) at room temperature for 2, 4, 6, 12, and 72 h. Then, the solutions were analyzed by ESI MS: we observed that in all the solutions, ions at *m*/*z* 171 of the protonated 3-aminomethyl-3,5,5-trimethylcyclohexylamine were detectable only after 6 h release and after 24 h release, they were also accompanied by ions at *m*/*z* 210 of the protonated 4,4′-methylenebis(cyclohexylamine). In the case of **2a** and **2b** solutions, ions at *m*/*z* 171 were abundant, representing the base peak after 24 hr. Thus, our hypothesis is that the antimicrobial activity, particularly the virucidal one, is linked to the release, in appropriate amounts, of 3-aminomethyl-3,5,5-trimethylcyclohexylamine and/or 4,4′-methylenebis(cyclohexylamine). To confirm this, detailed studies of the biological activity of these amines are underway. As a matter of fact, many amines of different chemical nature (i.e., aliphatic, aromatic, polyamines, quaternary ammonium salts) are endowed with marked antimicrobial properties [42,43,44]. It should be noted that the cytotoxicity tests indicate that the released molecules do not cause significant damage to the cells. The biological activity of cyclohexylamine derivatives has been reported in the literature [45,46,47]. In the case of resin **3**, no release of organic or inorganic compounds has been detected, and the presence of TiO_2_ as filler did not confer antimicrobial properties to the surface.

To better understand the antimicrobial properties shown by the epoxy resins under investigation, we studied both the physical and chemical properties of the surfaces. The determination of the point of zero charge gave the following results: 8.98 for **2a** > 8.06 for **3** > 7.45 for **1a**. These data indicated that at neutral pH, the surfaces exhibited a positive charge in the order **2a** > **3** > **1a**, thus indicating that bacteria bearing negatively charged head groups of acidic phospholipids in their membranes could strongly interact with the positively charged surfaces via electrostatic interaction, thus inducing damages similar to those induced by quaternary ammonium systems, which simultaneously disrupt the cell membrane integrity and deactivate the bacterial enzymes, leading to bacterial death [48] and thus behaving as a polycation system [49]. Similar consideration could be proposed for virus particles, whose distribution of charges is due to a distinct arrangement of the proteins in their structure, which differs for different types of viruses [50] and is responsible for their persistence on the surface of inanimate objects for days [51,52].

A preliminary evaluation of static contact angle for all the investigated samples gave values lower than 90°, thus indicating the hydrophilic nature of the surfaces [53]; not nanostructured [54] but behaving as a polycation bearing aminolkylamine moieties, for which antimicrobial activity has been demonstrated [55,56]. Thus, we could explain the antimicrobial activity of polymers **2a** and **2b** with a synergistic effect of active attack due to the release of killing agents and passive action to prevent biofouling formation. However, they cannot be considered “smart” antibacterial surfaces [57,58], whose properties can help to reduce the accumulation of dead bacteria and debris, which shields the functional groups, thus reducing bactericidal efficacy and facilitating subsequent bacterial adhesion.

We should remember that we are considering bulk materials and not simple coatings; thus, we can expect to achieve a self-sustained sterilizing effect, which could be of great importance in the production of objects with high tactility like those for hospital institutions, for which mechanical, wear resistance, and persistence of antimicrobial properties are necessary. The use of nanocomposites in dentistry is analogous, for which a combination of antimicrobial and favorable mechanical properties is essential [59], even if the addition of nanoparticles can affect the dissemination of antibiotic resistance in bacteria; this highlights the potential hazard of introducing nanomaterials into the environment without a complete understanding of their wider consequences.

## 5. Conclusions

Three commercial epoxy resins, **1** (EC157/W152LR), **2** (MGS/RIMH235), and **3** (MC152/EW101), which serve as fundamental base materials for the design and realization of multifunctional nanocomposites, were investigated in vitro to ascertain their antimicrobial activity. Resins **1** and **2** demonstrated a relevant antibacterial activity against *E. coli* and *S. aureus*, with resin **2** also exhibiting a significant virucidal activity against two human pathogenic viruses transmitted by salivary droplets. These experiments were conducted on bulk samples, not just coatings, suggesting that they could withstand cleaning processes carried out on their surfaces, potentially renewing their antimicrobial efficacy.

The insights gained from this study have significant implications across diverse fields, including material science and engineering, public health, and environmental protection. By harnessing the synergistic combination of mechanical strength and antimicrobial functionality, epoxy-based composites have the potential to revolutionize the design and performance of advanced materials, paving the way for innovative solutions to combat microbial threats and enhance the sustainability of critical infrastructure.

In summary, the results reported in this paper, combining the mechanical and antimicrobial properties exploration of extensively used commercial epoxy resins, not only give an understanding of the underlying principles of their behavior but also suggest the practical implications of their application in future scenarios. The chemical–physical and biological data made available can be the starting point for the design and realization of new “smart” objects based on these materials, which are able to maintain their antimicrobial properties over time.

Considering the recent struggle to deal with widespread infections such as COVID-19 and the risk posed by those we will face in the foreseeable future; the development and commercial availability of these materials is of paramount importance. These epoxy resins are deemed suitable to contribute to limiting the spread of infection, particularly in confined locations and situations that are more susceptible to microbial contamination, such as medical clinics and hospitals.

Nonetheless, the durability and long-term stability of the antimicrobial properties under different environmental conditions needs to be investigated to assess the robustness and practical applicability of these materials and their design derivatives.

## Figures and Tables

**Table 1:** Samples labeling and selected experimental and characterization data.

Sample	Epoxy Resin	Resin Precursor Amount (g)	Curing Agent Amount (g)	Density (g/cm^3^) ^1^	T_g_ (°C) ^2^	TGA (°C) ^3^
**1a**	EC157/W152LR	120	40	1.153	79.9 (74)	332
**1b**	EC157/W152LR	120	45	1.148	82.9 (84)	330
**2a**	MGS/RIMH235	100	34	1.156	79.9 (85)	338
**2b**	MGS/RIMH235	100	38	1.160	80.9 (80)	333
**3**	MC152/W101	100	12	1.797	48.6 (53)	319 ^4^

^1^ The values are the average of three independent measurements on five different samples. ^2^ The values were obtained by DMA determinations (loss modulus); in parentheses, the values obtained by DSC analyses (cycle 3). ^3^ T_onset_ according to ISO 11358-1 [29]. ^4^ A second thermal degradation with a weight loss of 26% at 709 °C due to the carbonates’ decarboxylation.

**Table 2:** Mechanical properties determined during testing. Average values and standard deviations.

Sample	Tensile Strength (MPa)	Young Modulus (GPa)	Fracture Toughness (MPa m^0.5^)
**1a**	71.2 ± 3.0	2.86 ± 0.13	0.90 ± 0.09
**1b**	59.1 ± 3.1	2.59 ± 0.25	1.17 ± 0.14
**2a**	54.4 ± 0.6	2.42 ± 0.29	1.31 ± 0.13
**2b**	67.6 ± 1.2	2.87 ± 0.06	1.04 ± 0.11
**3**	26.7 ± 0.7	8.87 ± 0.65	2.60 ± 0.18

## Data Availability

The original contributions presented in the study are included in the article/Supplementary Materials; further inquiries can be directed to the corresponding authors.

## Supplementary Materials

The following supporting information can be downloaded at: https://www.mdpi.com/article/10.3390/polym16182571/s1, Figure S1: FT IR and ^1^HNMR of the epoxy precursors for the preparation of samples **1**–**3**; Figure S2: FT IR and ^1^HNMR of the hardeners for the preparation of samples **1**–**3**; Figure S3: TGA, DSC, and DMA determinations of samples **1**–**3**; Figure S4: Selected ESEM and EDX data for samples **1**–**3**; Table S1: EDX data for samples **1**–**2**.
