# Supplementary material for: Revealing Commercial Epoxy Resins’ Antimicrobial Activity: A Combined Chemical–Physical, Mechanical, and Biological Study
Source: Polymers (Basel). 2024 Sep 11;16(18):2571. doi: 10.3390/polym16182571 (PMC11435071; doi:10.3390/polym16182571)
Supplement: Supplementary file 1 [file polymers-16-02571-s001.zip › polymers-3165137-supplementary.pdf]

SUPPLEMENTARY INFORMATION

Revealing commercial epoxy resins' antimicrobial activity: a combined chemical-physical, mechanical, and biological study

Mario Rigo, Hamoun Khatami, Antonella Mansi, Anna Maria Marcelloni, Anna Rita Proietto, Alessandra Chiominto, Ilaria Amori, Annalisa Bargellini, Isabella Marchesi, Giuseppina Frezza, Francesco Lipani, Claudio Cermelli, Angelo Rossini, Marino Quaresimin, Michele Zappalorto, Alessandro Pontefisso, Matteo Pastrello, Daniele Rossetto, Michele Modesti, Paolo Sgarbossa, Roberta Bertani

Figures S1: **FT IR and ^{1}HNMR of the epoxy precursors for the preparation of samples 1-3**

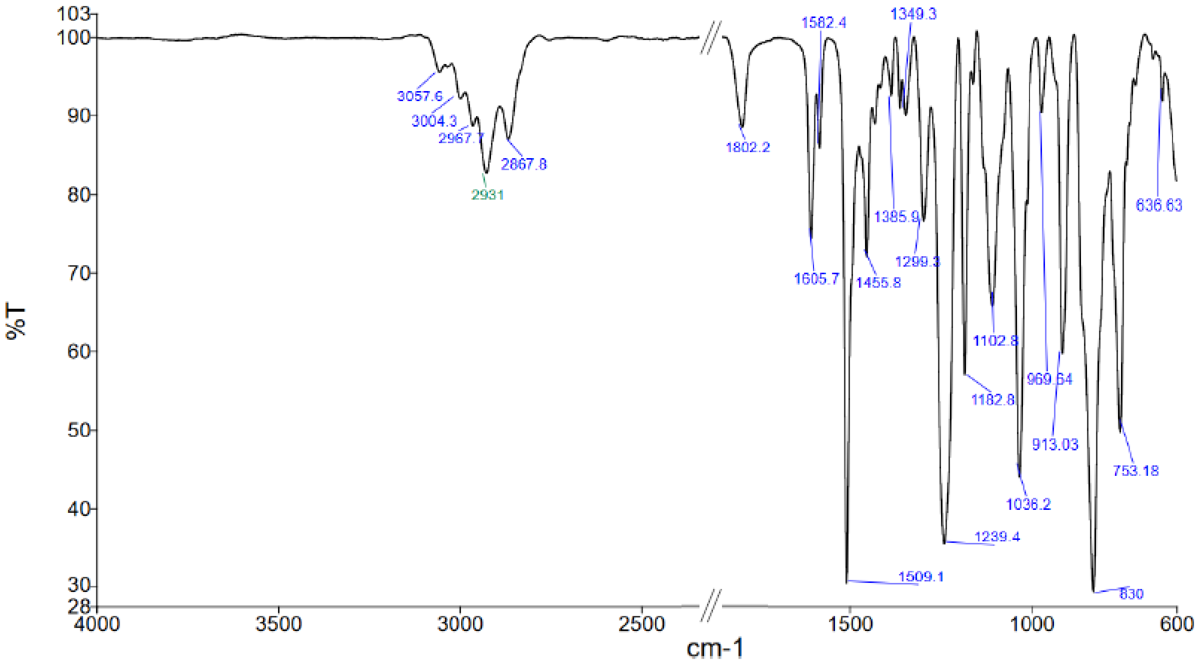

Figure S1a: FTIR spectrum neat of the epoxy precursor DGEBA, Elan Tech EC157

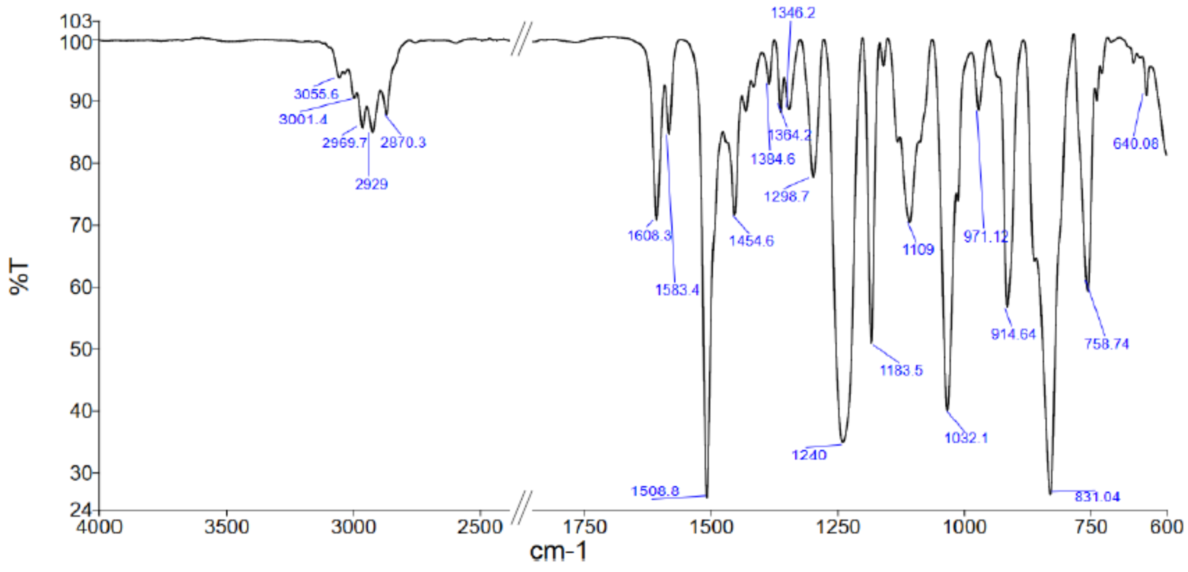

Figure S1b: FTIR spectrum of the neat epoxy precursor EPIKOTE™ Resin MGS®

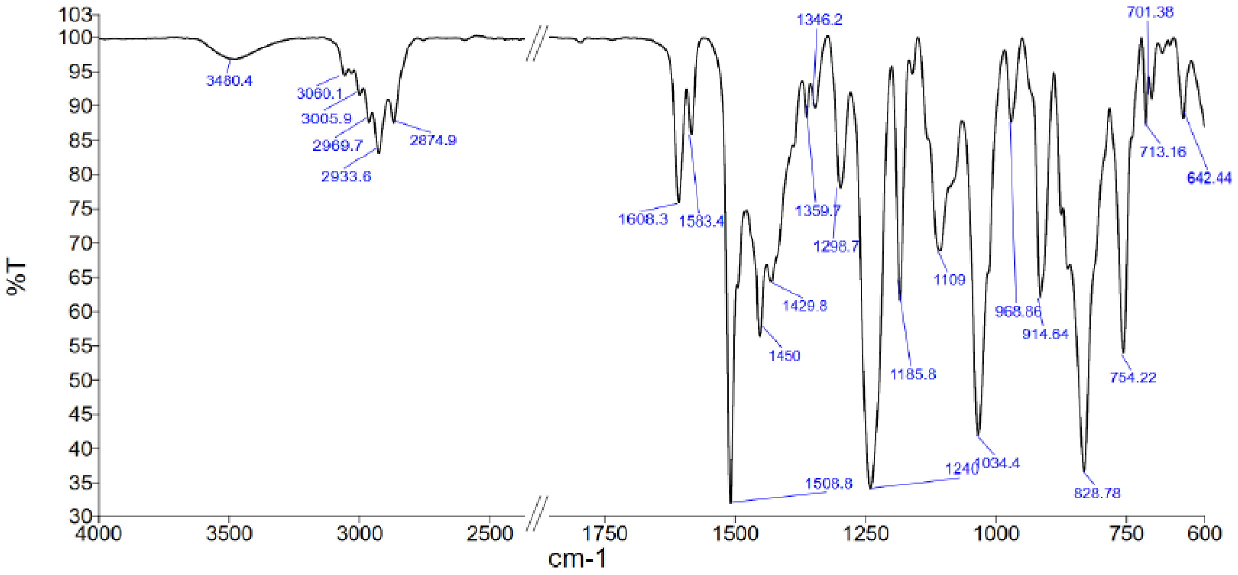

Figure S1c: FTIR spectrum of the neat epoxy precursor MC152

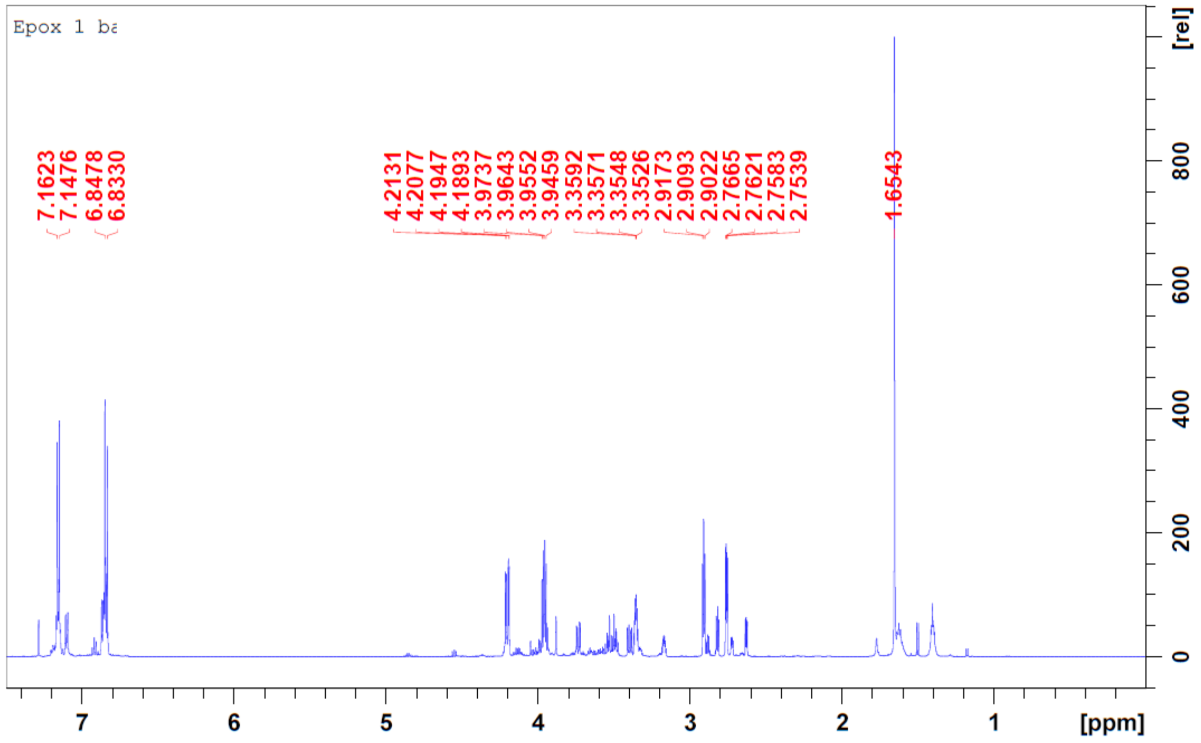

Figure S1d: ^{1}H NMR spectrum of the epoxy precursor DGEBA, Elan Tech EC157 in $CDCl_3$

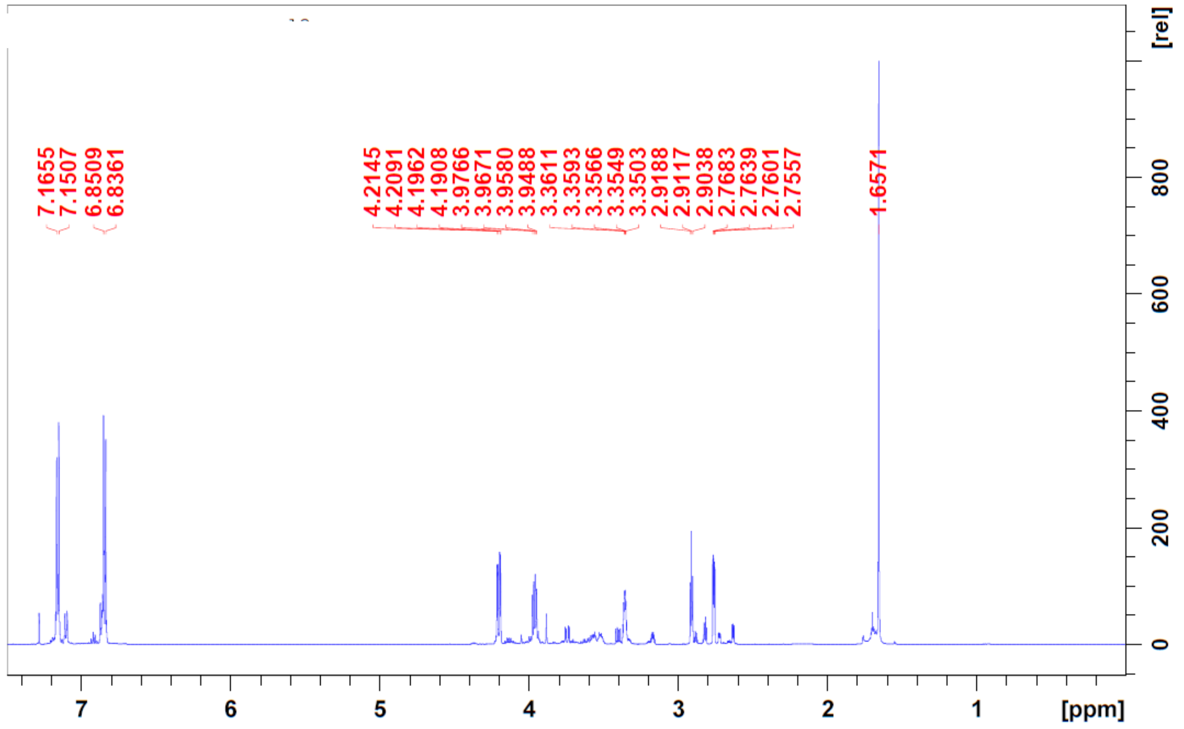

Figure S1e: ^{1}H NMR spectrum of the epoxy precursor EPIKOTE^{TM} Resin MGS® in $CDCl_3$

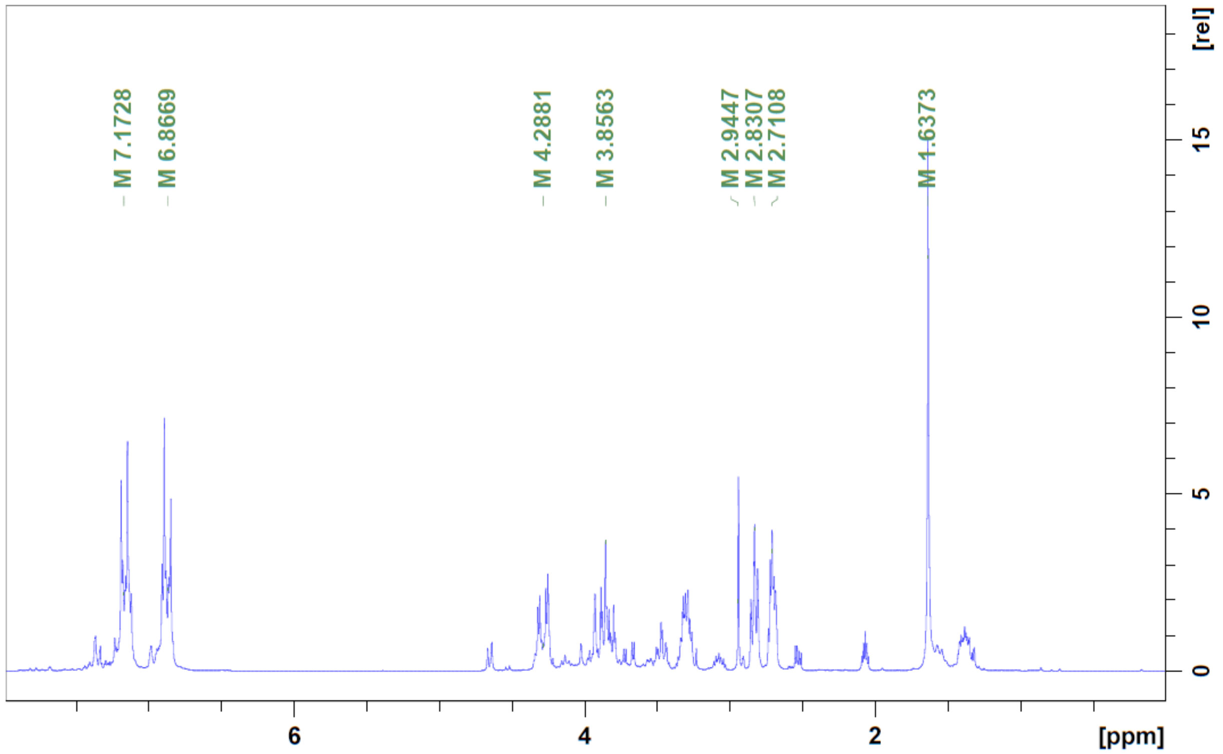

Figure S1f: ^{1}H NMR spectrum of the epoxy precursor MC152 in CD_3COCD_3

The epoxy resins are mixtures of

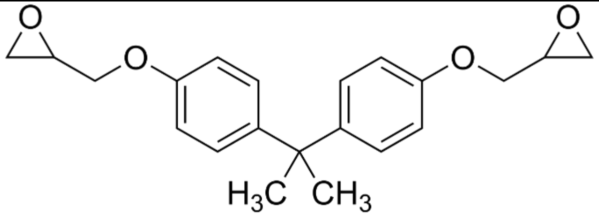	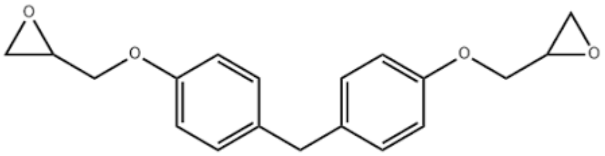
bis-phenol-A-epichlorydrin and	bis-phenol-F-epichlorydrin

Figures S2: **FT IR and ^{1}HNMR of the hardeners for the preparation of samples 1-3**

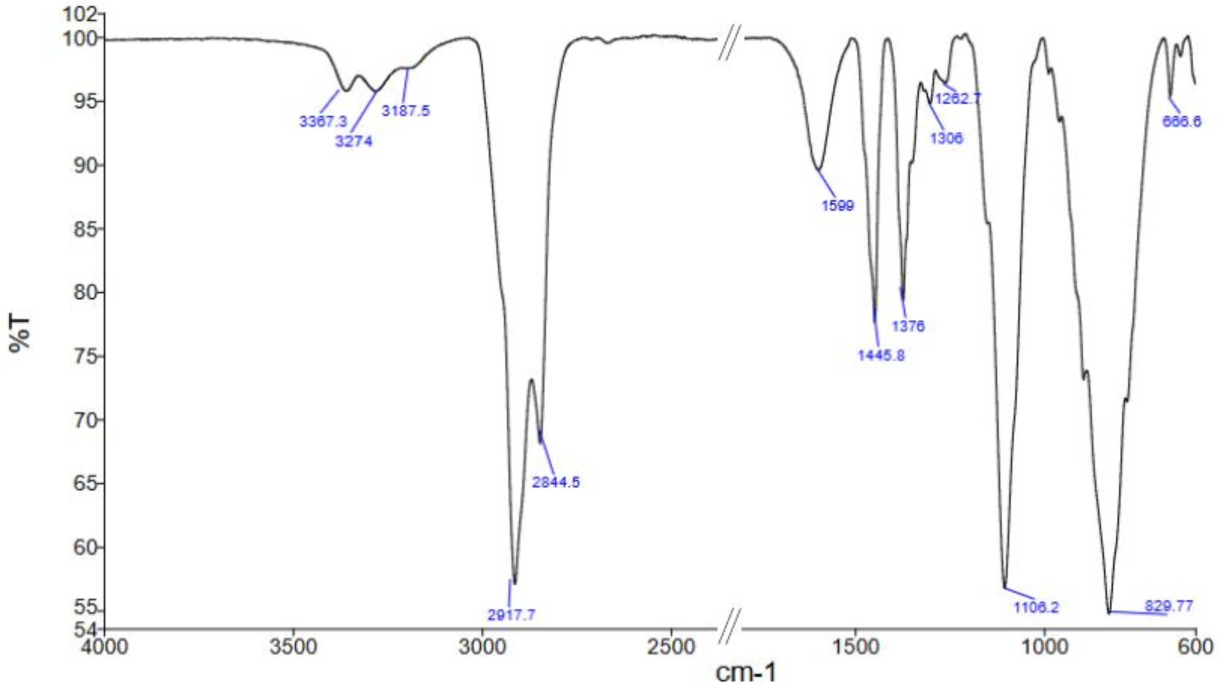

Figure S2a: FTIR spectrum of the neat hardener Elan-TechW 152LR

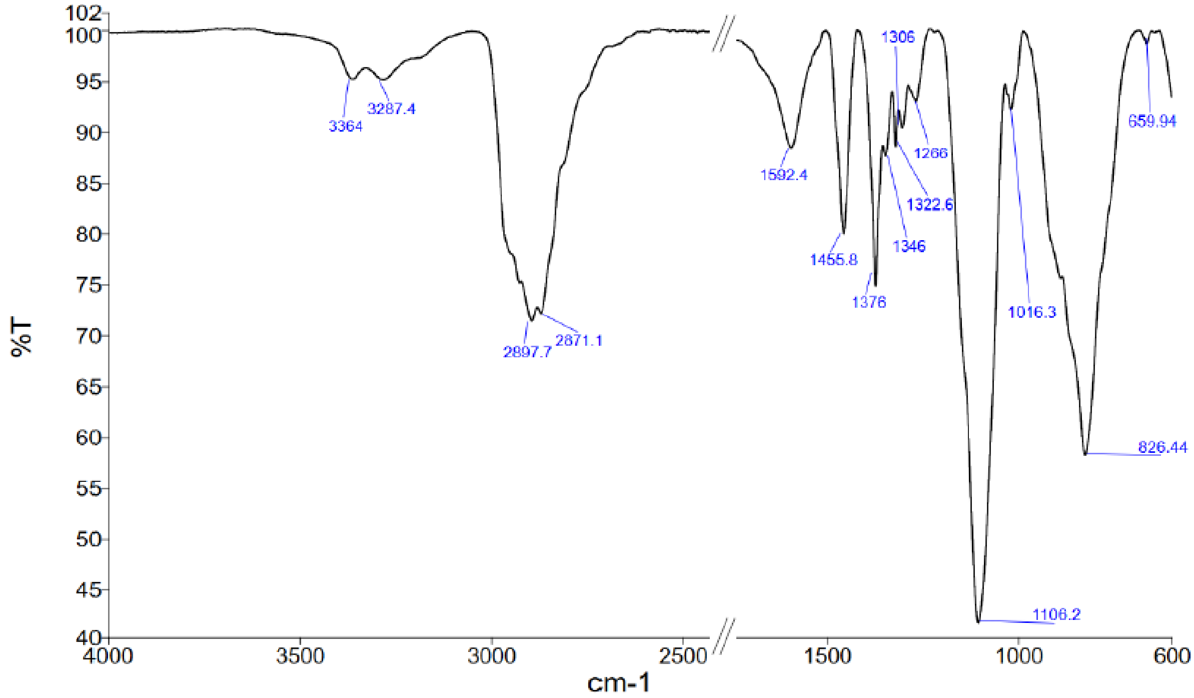

Figure S2b: FTIR spectrum of the neat hardener EPIKURE^{TM} RIM H 235

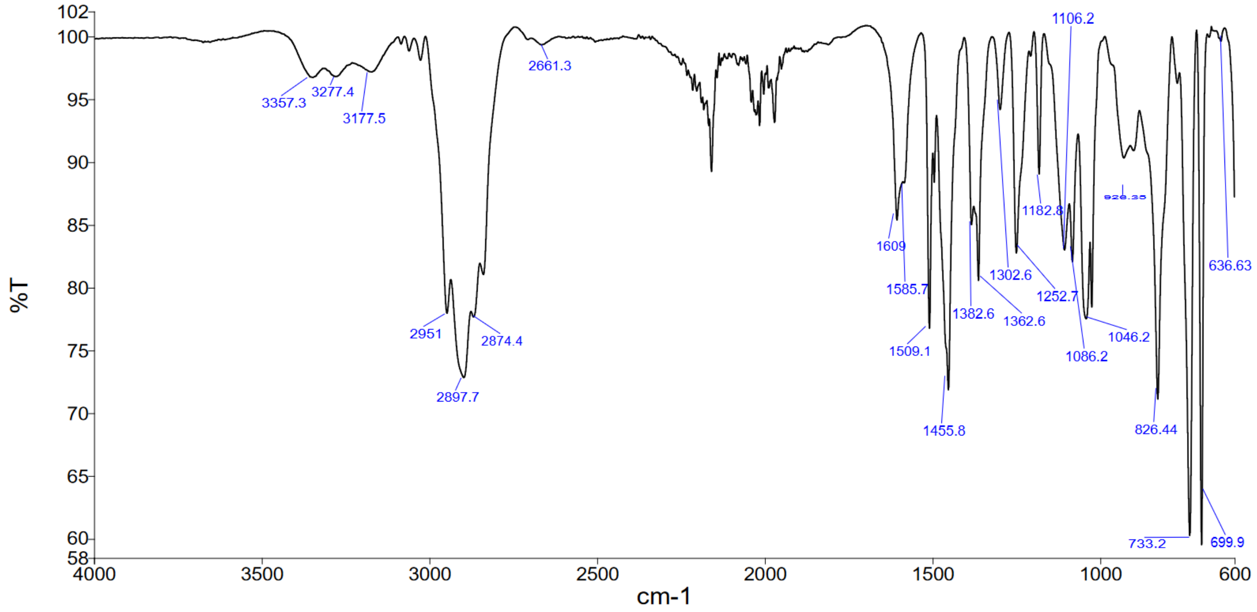

Figure S2c: FTIR spectrum of the neat hardener W101

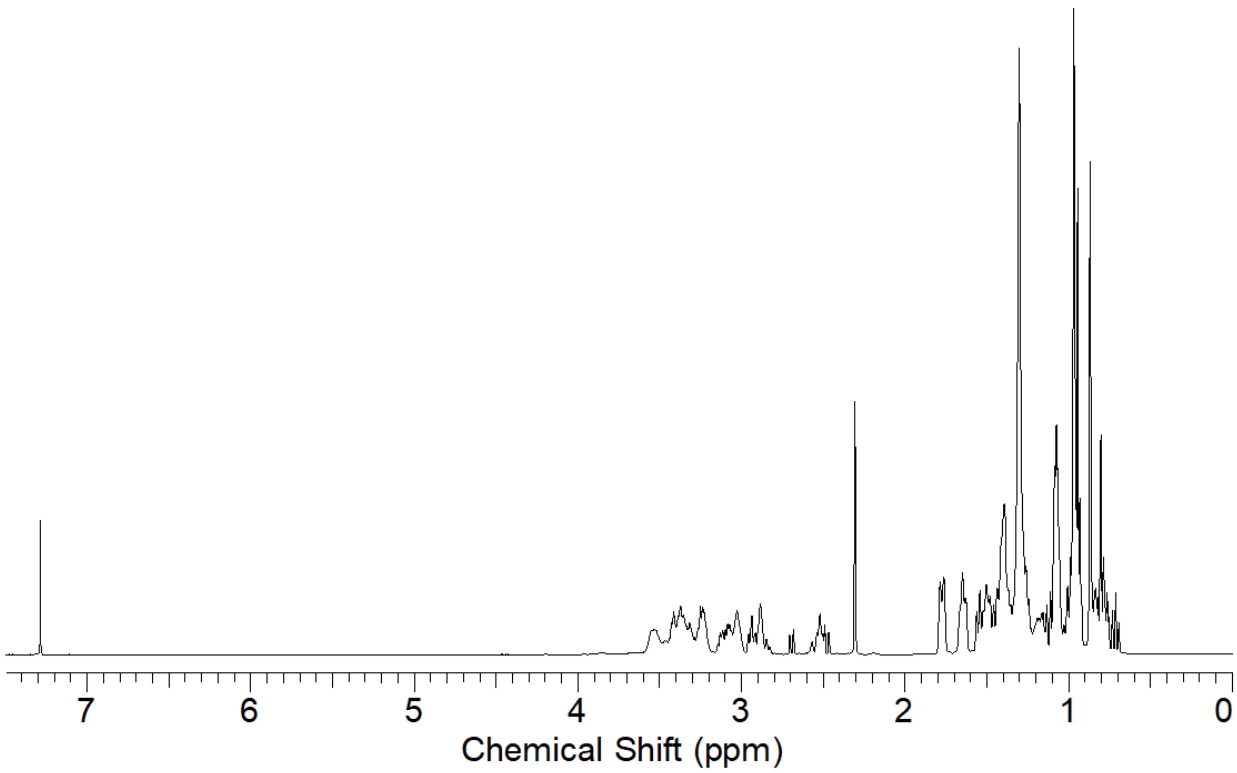

Figure S2d: ^{1}H NMR spectrum of the hardener Elan-TechW 152LR in $CDCl_3$

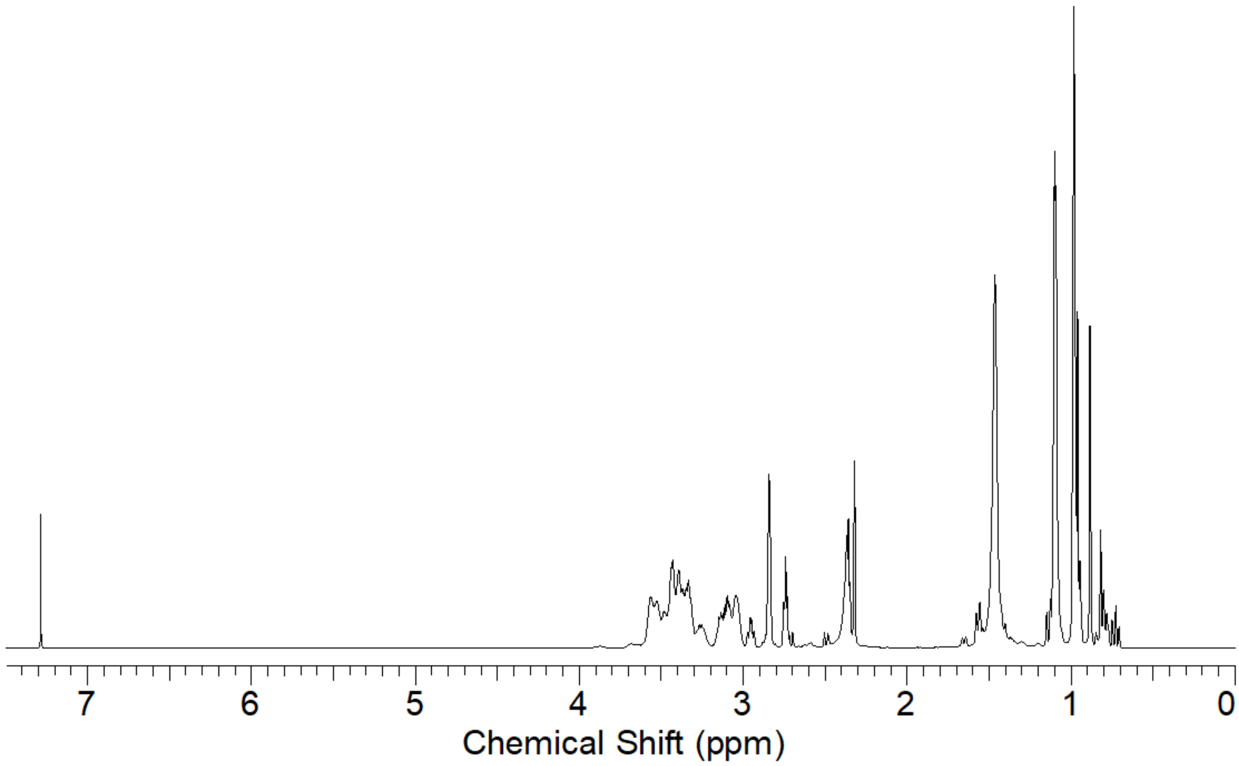

Figure S2e: ^{1}H NMR spectrum of the hardener EPIKURE^{TM} RIM H 235 in $CDCl_3$

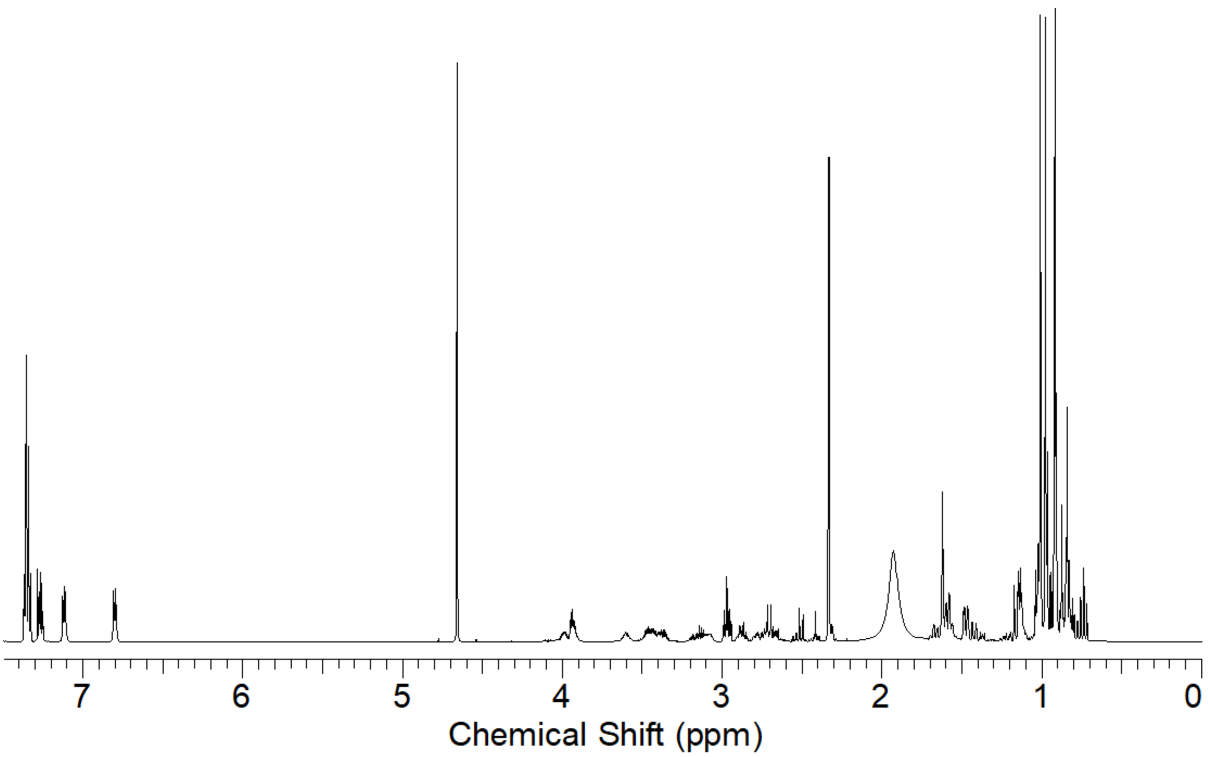

Figure S2f: ^{1}H NMR spectrum of the hardener W101 in $CDCl_3$

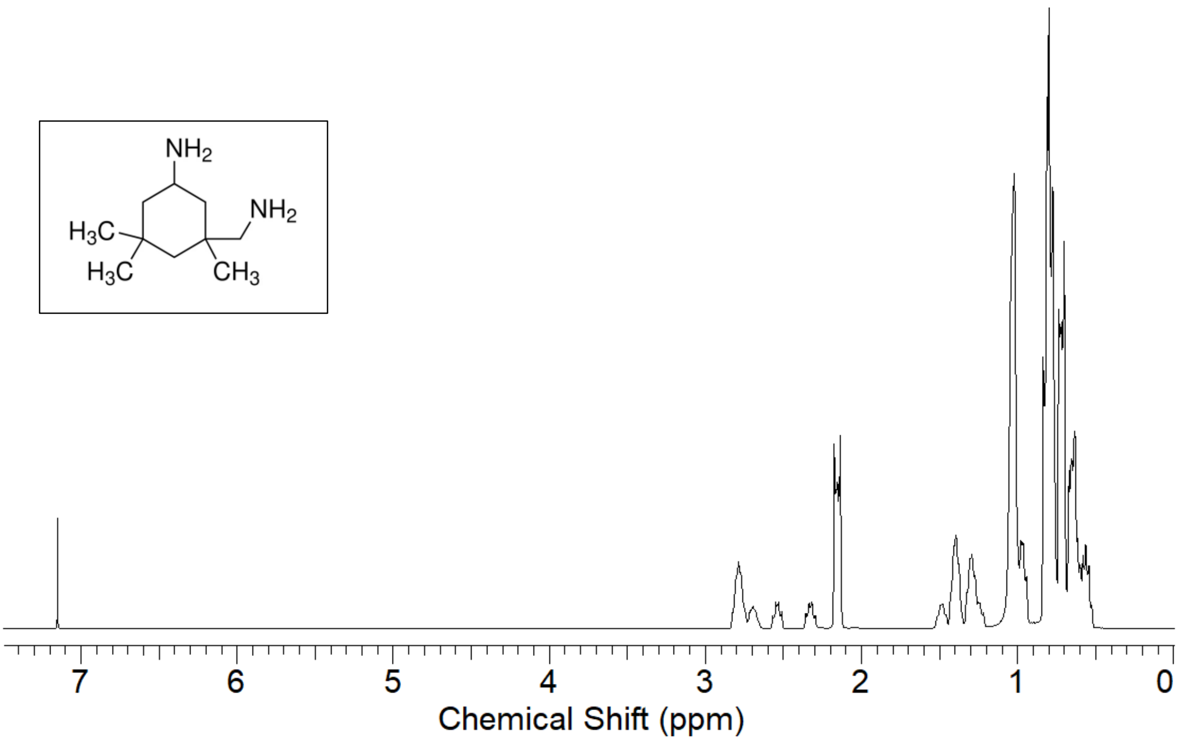

Figure S2g: ^{1}H NMR spectrum of 3-aminomethyl-3,5,5-trimethylcyclohexylamine in $CDCl_3$

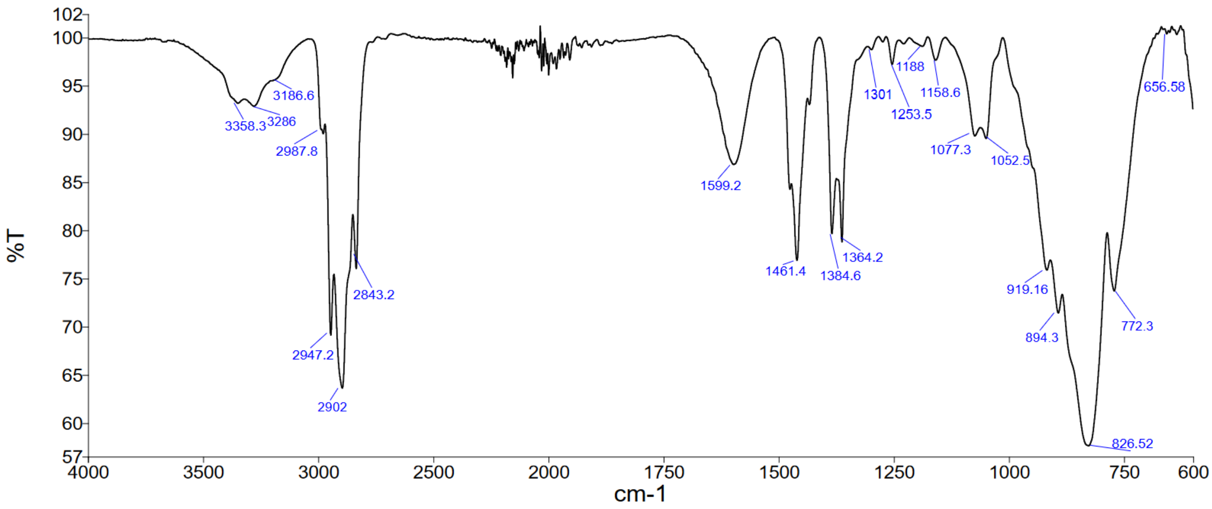

Figure S2h: FTIR spectrum of neat 3-aminomethyl-3,5,5-trimethylcyclohexylamine

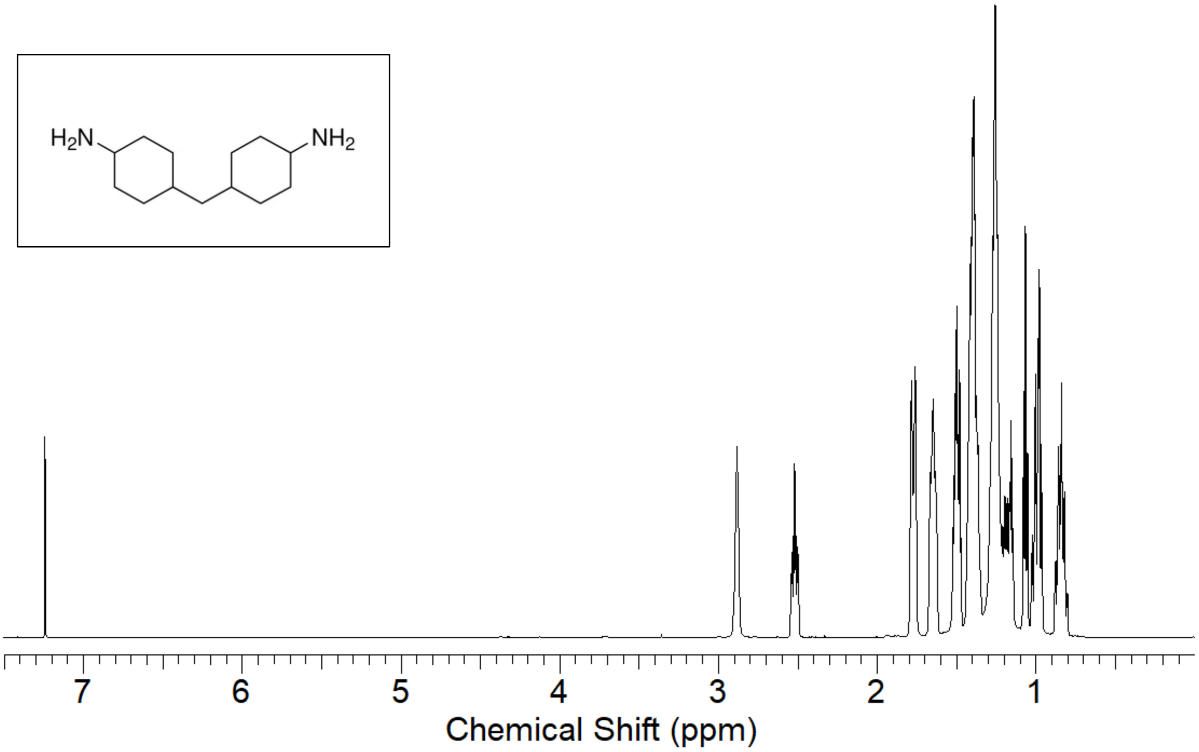

Figure S2i: ^{1}H NMR spectrum of 4,4'-methylenebis(cyclohexylamine) in $CDCl_3$

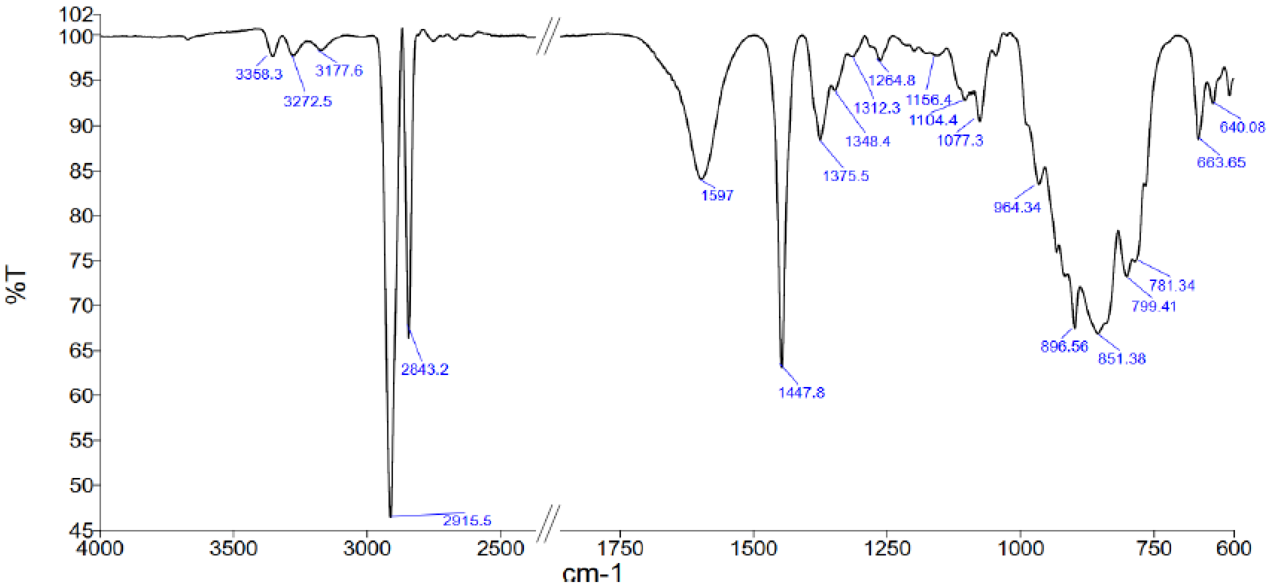

Figure S2j: FTIR spectrum of neat 4,4'-methylenebis(cyclohexylamine)

Figures S3: TGA, DSC, and DMA determinations of samples 1- 3

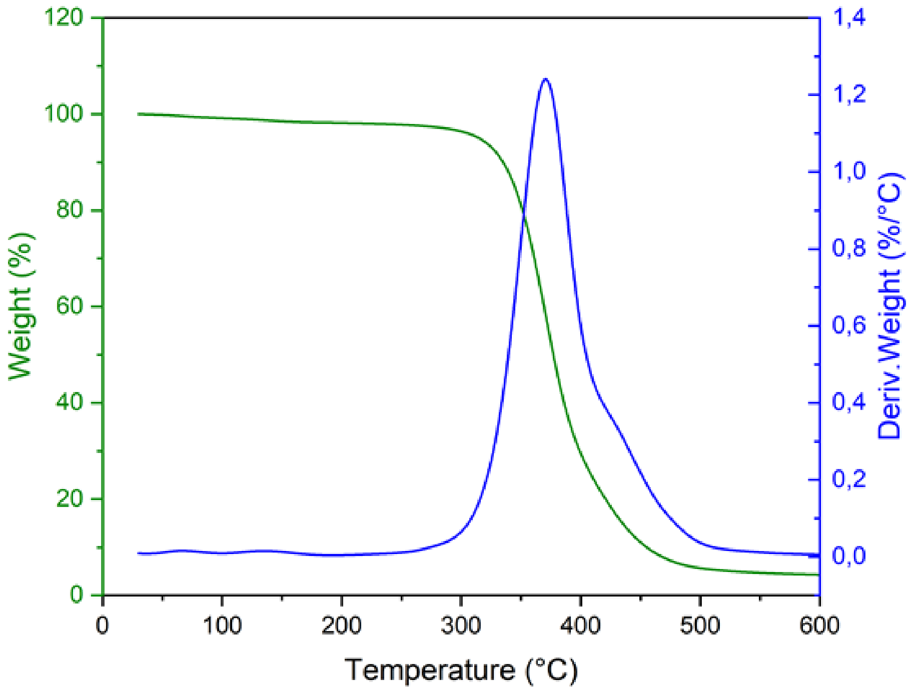

Figure SI 3a: TGA of epoxy resin **1a**

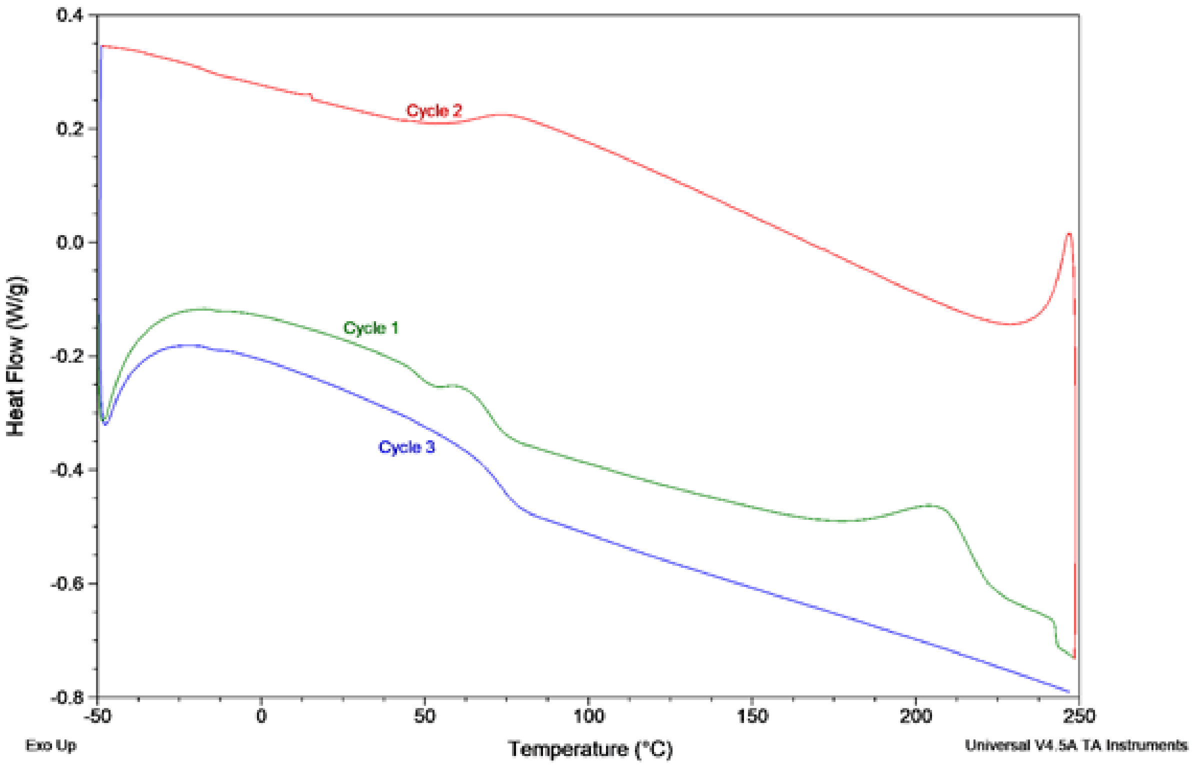

Figure S3b: DSC analysis of epoxy resin **1a**

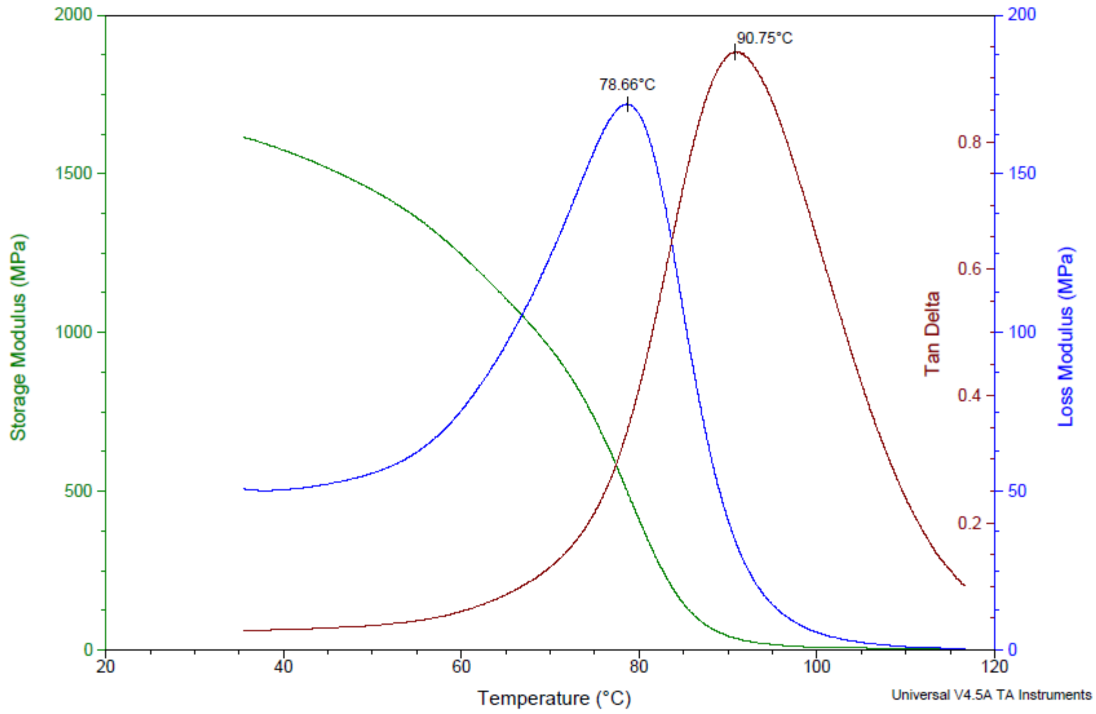

Figure S3c: DMA of epoxy resin **1a**

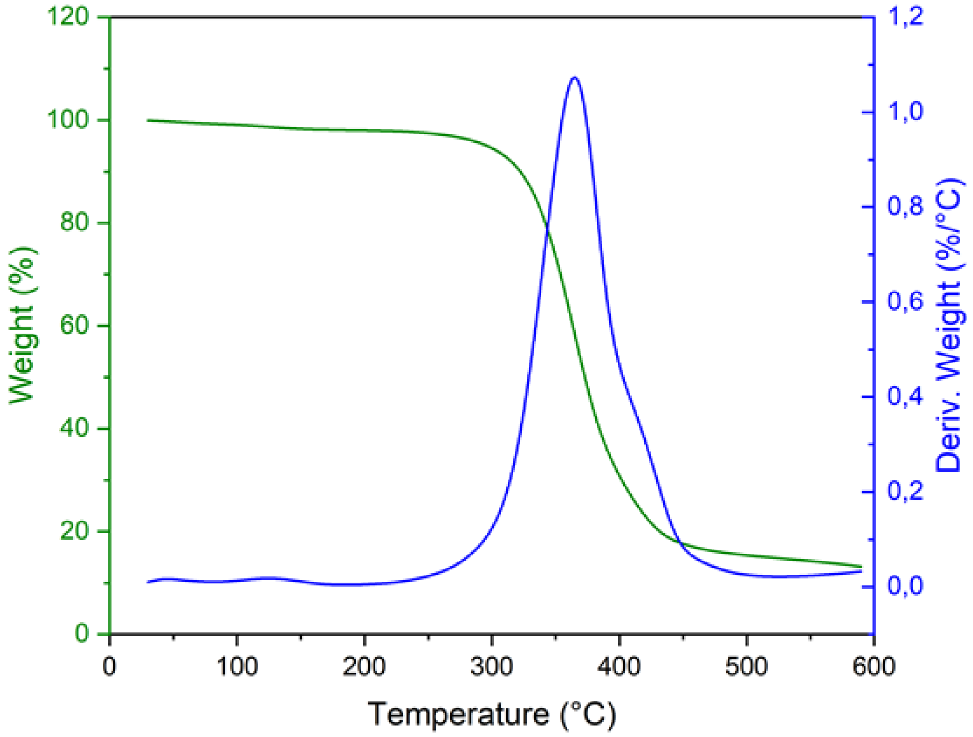

Figure S3d: TGA of epoxy resin **1b**

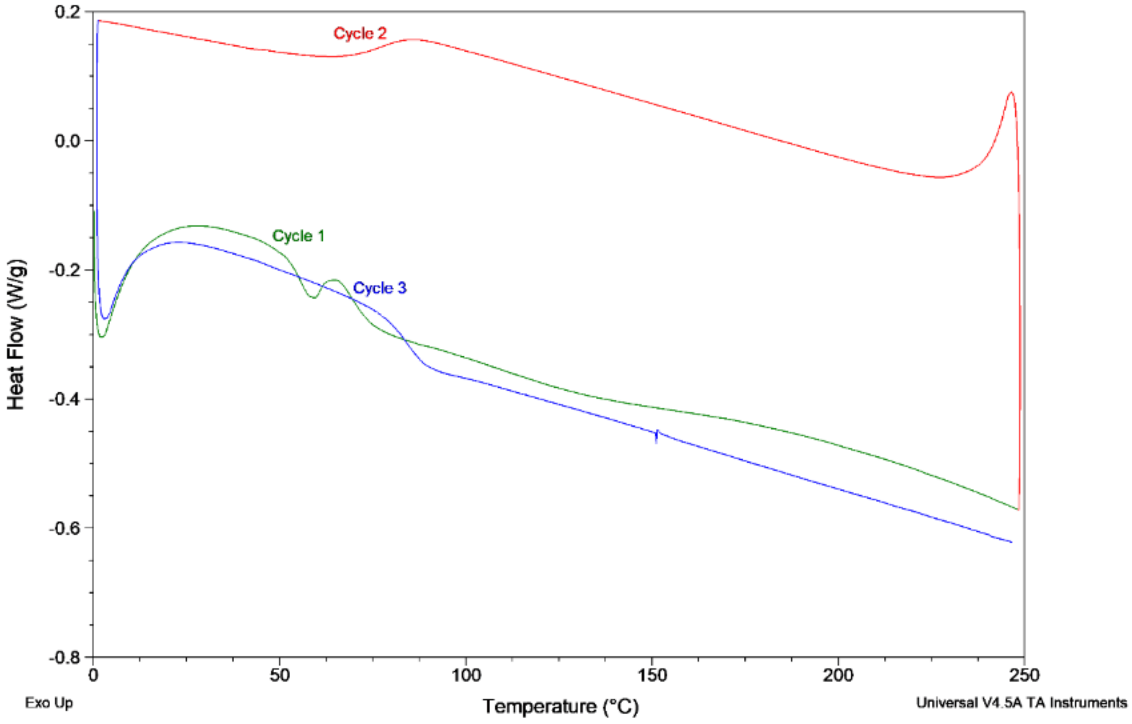

Figure S3e: DSC analysis of epoxy resin **1b**

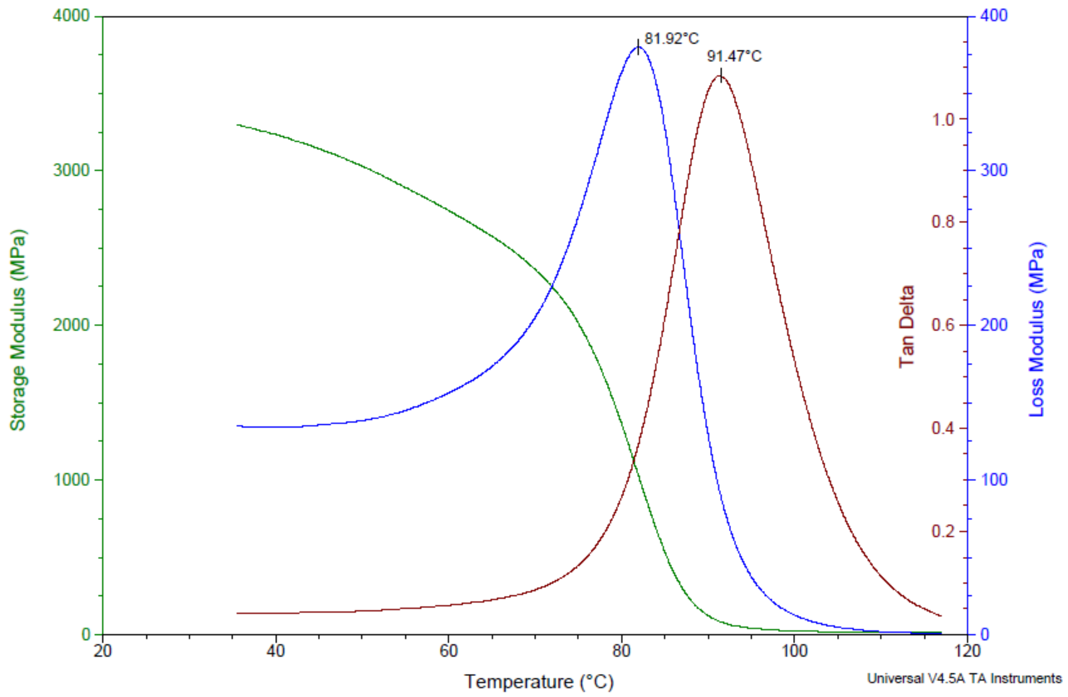

Figure S3f: DMA of epoxy resin **1b**

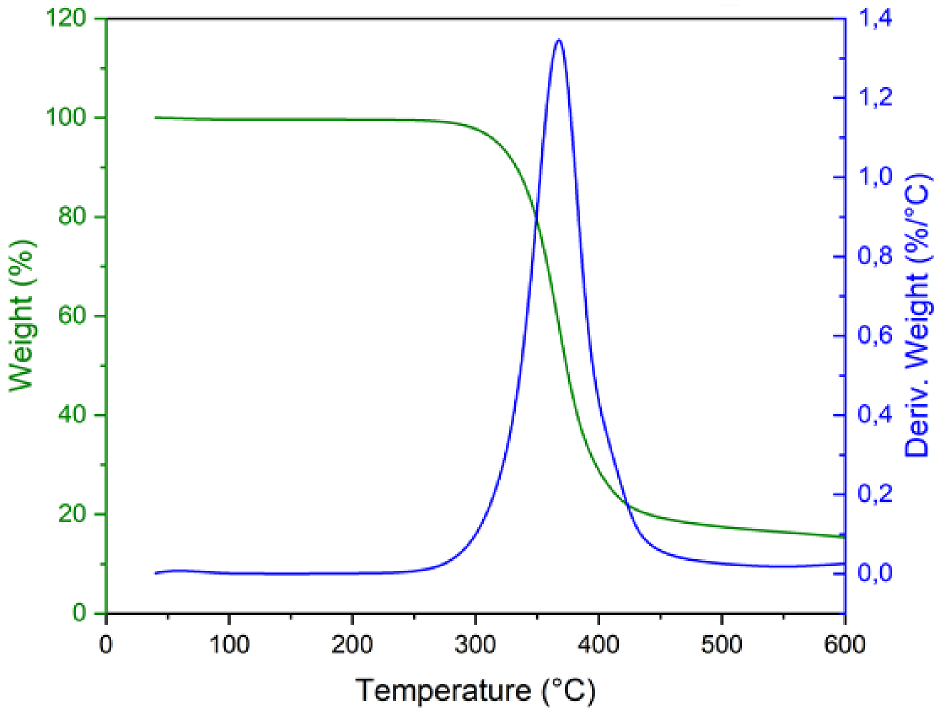

Figure S3g: TGA of epoxy resin **2a**

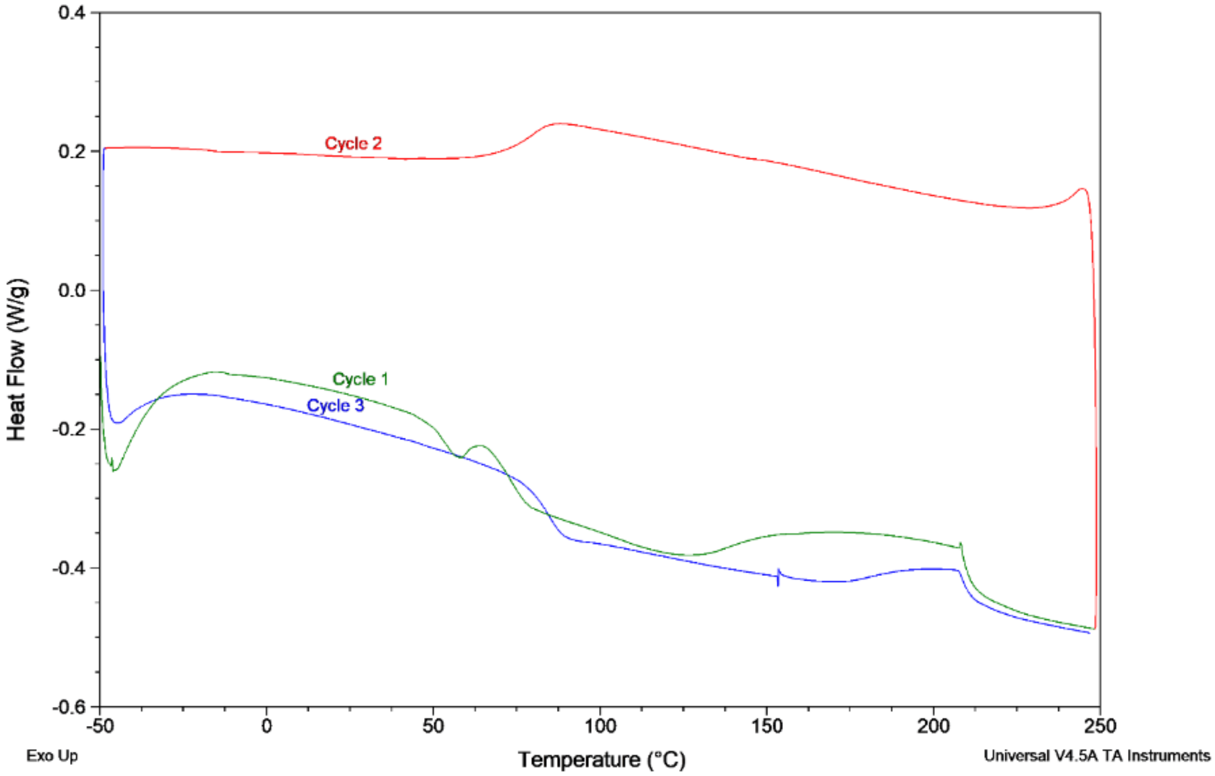

Figure S3h: DSC analysis of epoxy resin **2a**

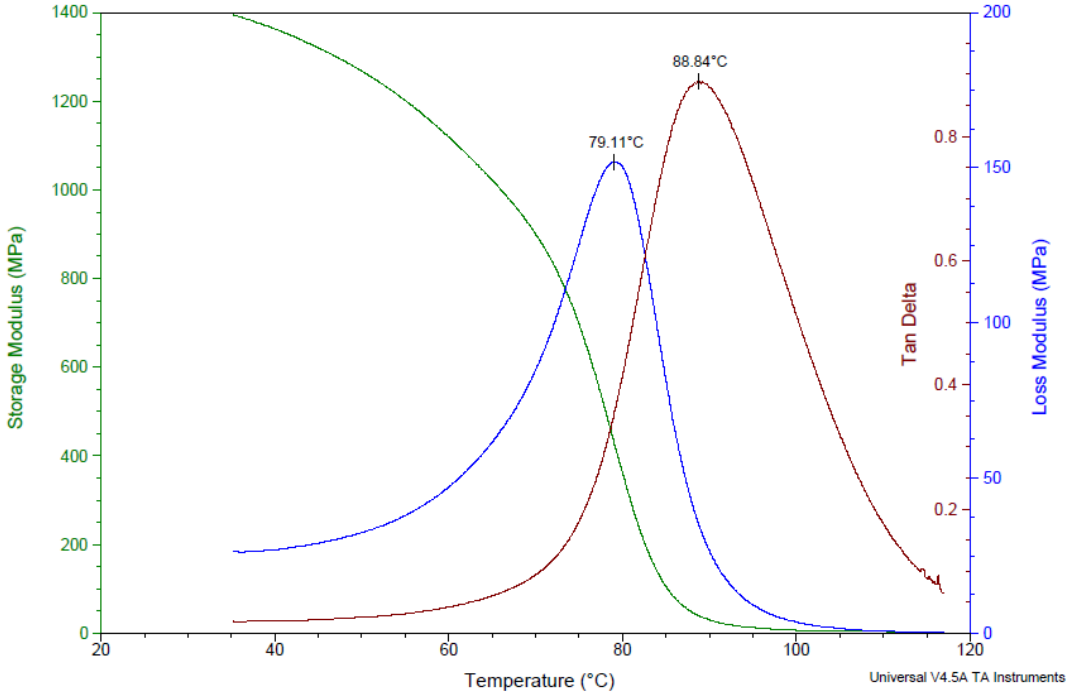

Figure S3i: DMA of epoxy resin **2a**

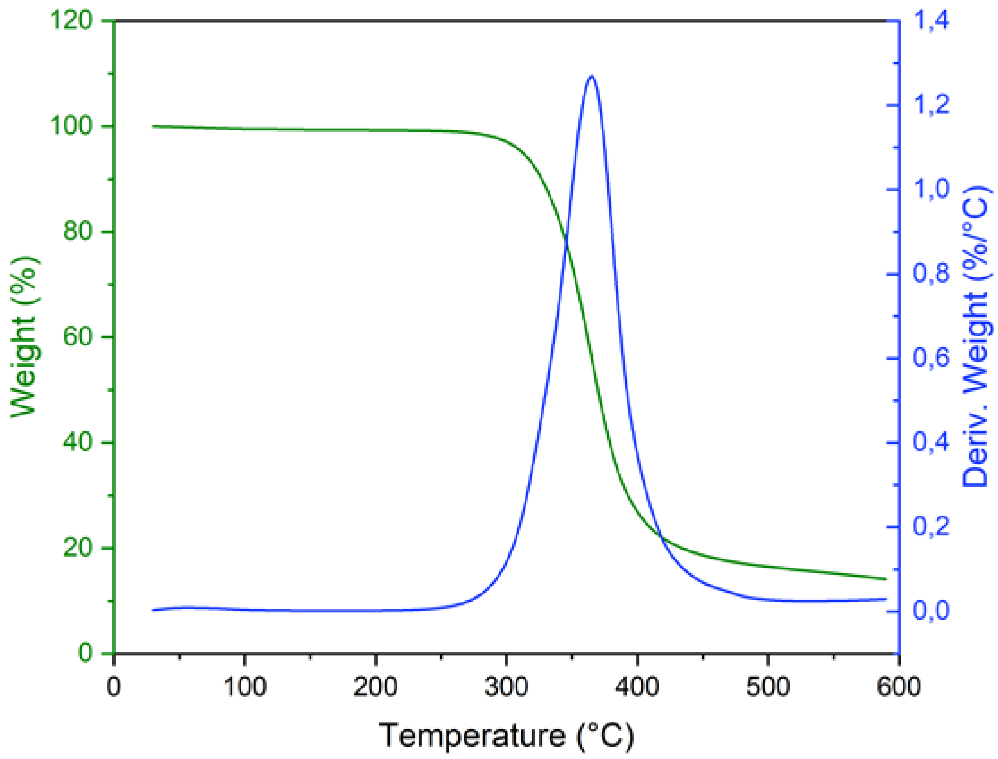

Figure S3j: TGA of epoxy resin **2b**

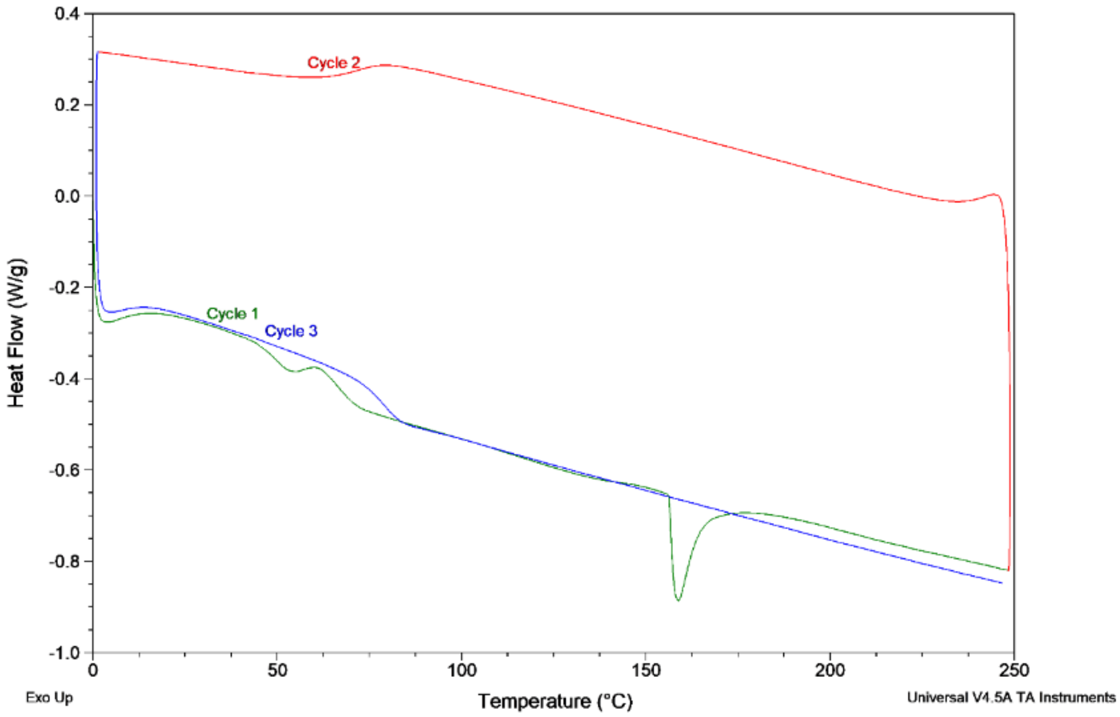

Figure S3k: DSC analysis of epoxy resin **2b**

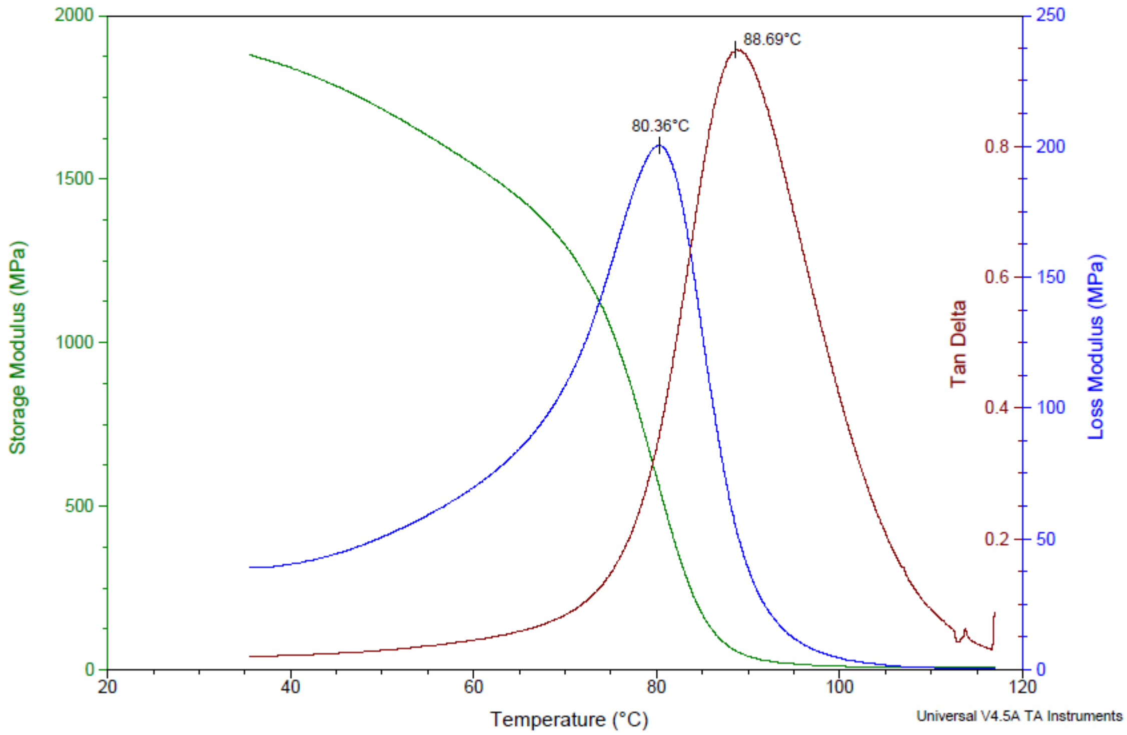

Figure S3l: DMA of epoxy resin **2b**

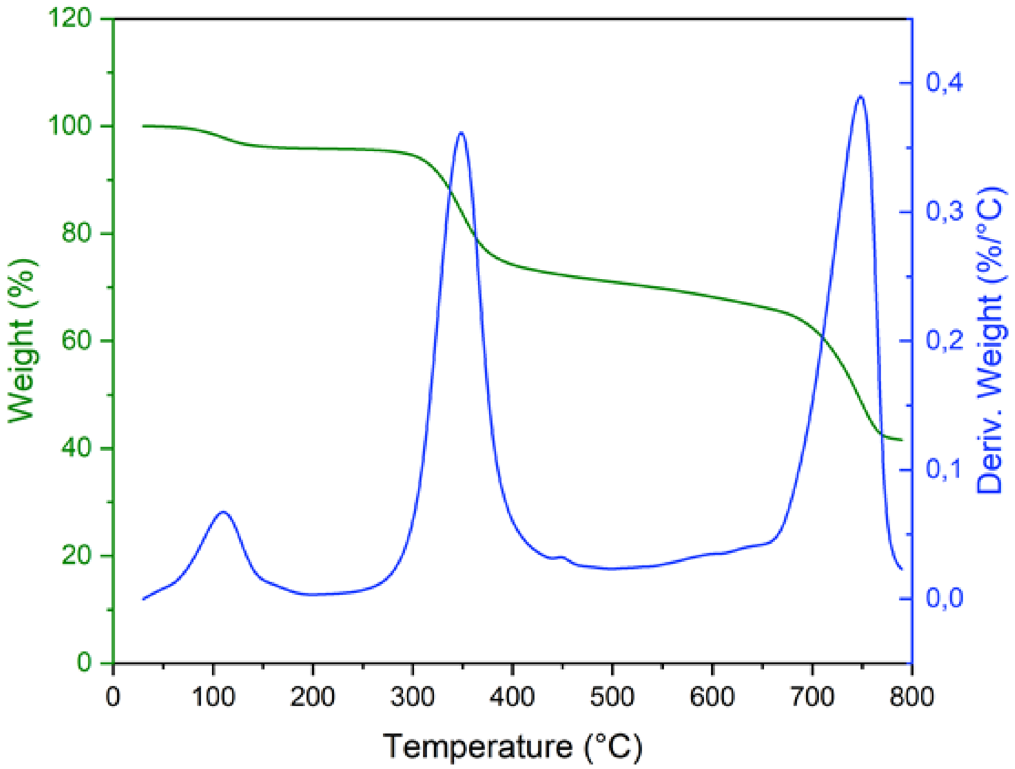

Figure S3m: TGA of epoxy resin **3**

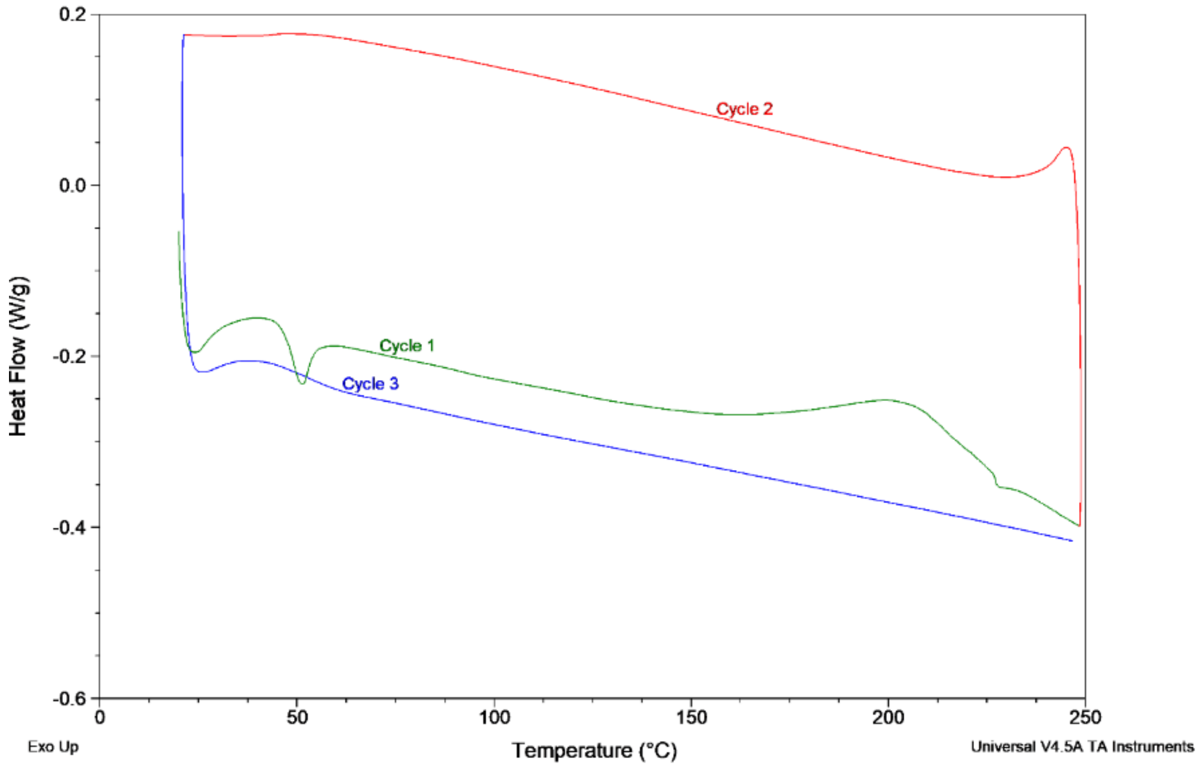

Figure S3n: DSC analysis of epoxy resin **3**

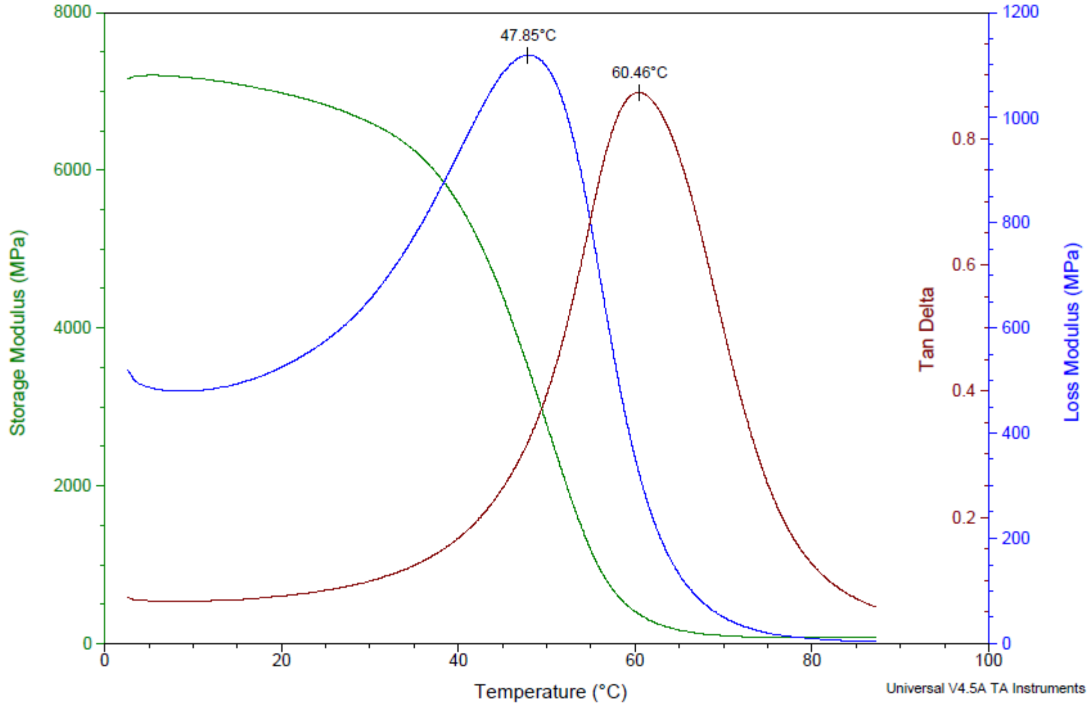

Figure S3o: DMA of epoxy resin **3**

Figures S4: Selected ESEM and EDX data for samples 1-3

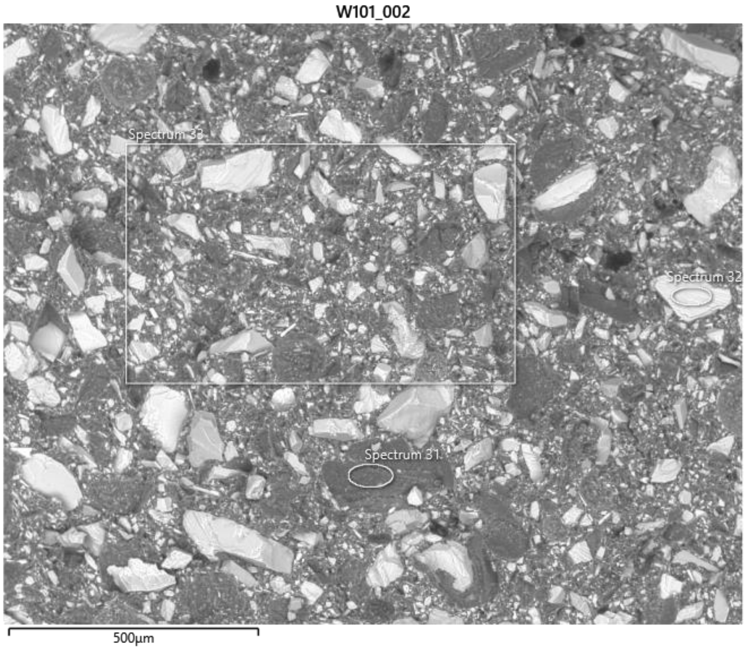

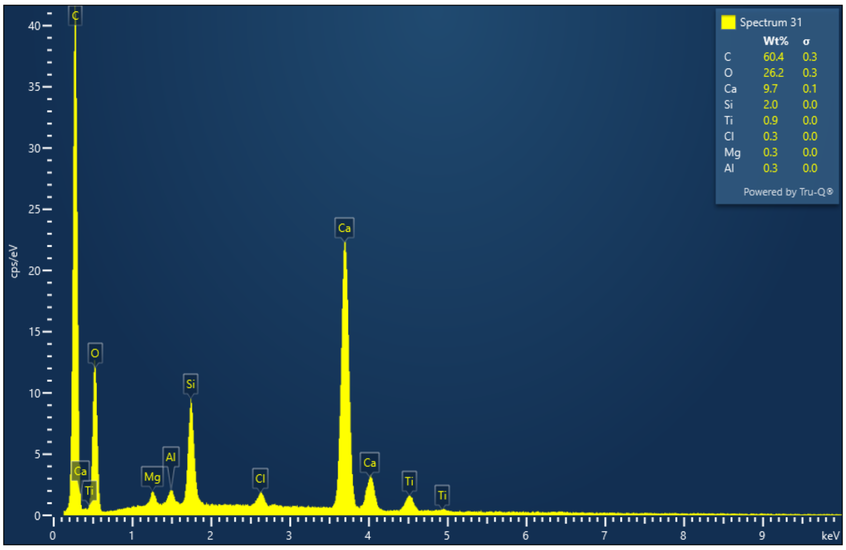

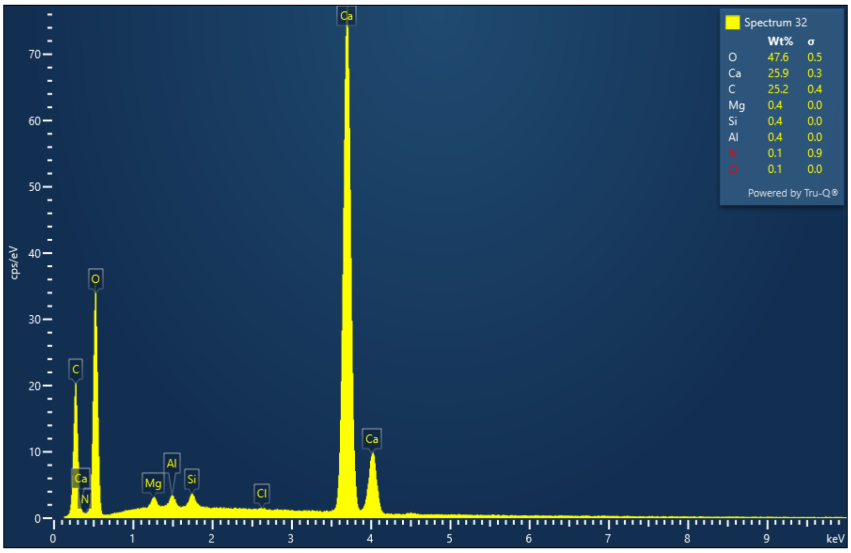

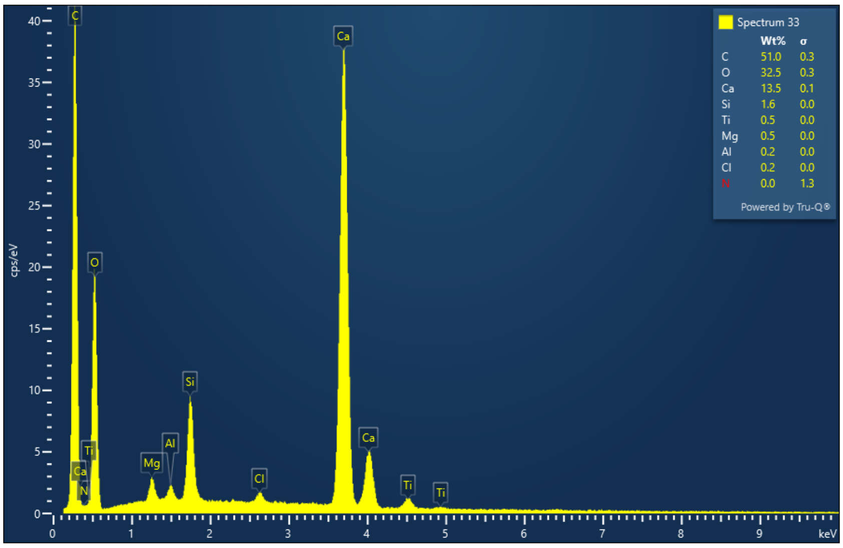

Figure S4a: ESEM and EDX data for resin MC152 and W101

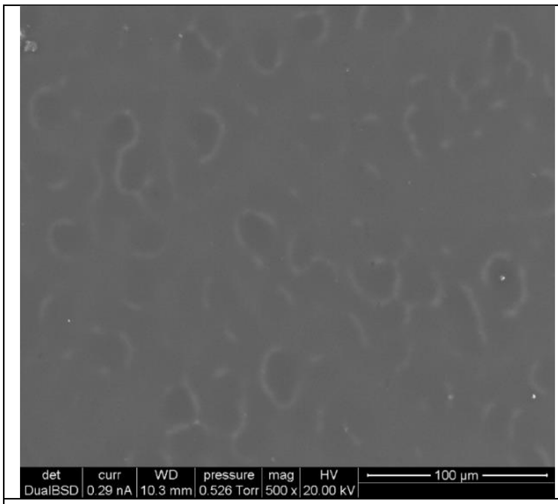

Image at 500X of **1a**

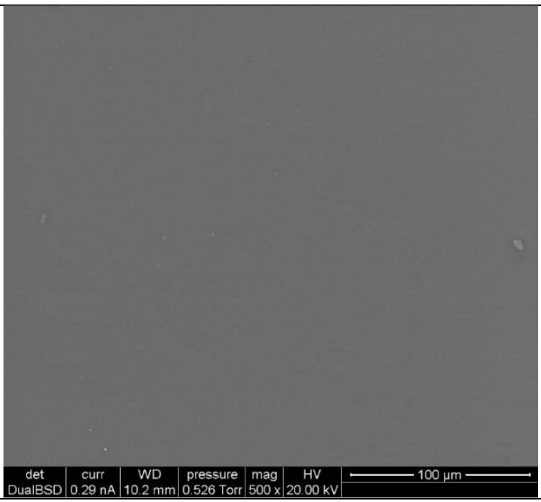

Image at 500X of **1b**

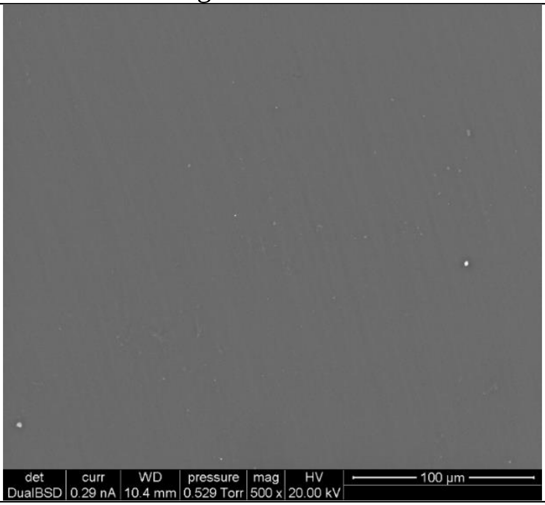

Image at 500X of **2a**

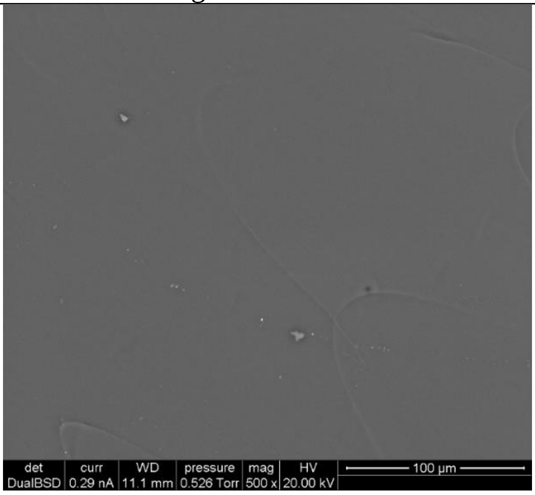

Image at 500X of **2b**

Figure S4b: ESEM images and data for resins **1** and **2.** The images refer to the surfaces obtained from the brittle fracture of the samples. the surfaces of the samples are homogeneous and the white dots you see are grains of dust necessary to bring the images into focus.

Table S1: EDX data for the samples **1-2**. The data (% w/w) are the average of five independent measurements on different specimens

element	**Sample 1a**	**Sample 1b**	**Sample 2a**	**Sample 2b**
C	79.4	78.4	79.1	78.8
O	17.6	18.0	19.2	19.3
N	2.2	2.4	1.0	1.2

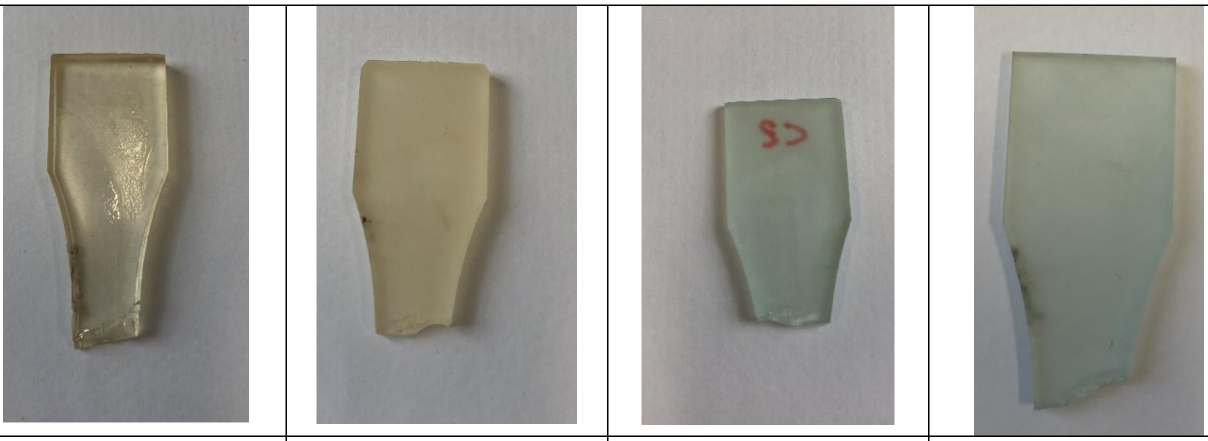

**Sample 1a**	**Sample 1b**	**Sample 2a**	**Sample 2b**

Figure S4c: Fragments of dog-bones of samples 1-2 after mechanical determinations

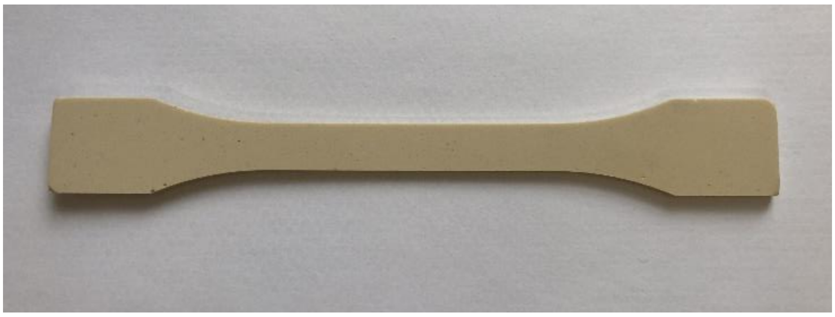

Figure S4d: Dog-bone for mechanical tests of sample **3**
